# Persistent mutation burden drives sustained anti-tumor immune responses

**DOI:** 10.1038/s41591-022-02163-w

**Published:** 2023-01-26

**Authors:** Noushin Niknafs, Archana Balan, Christopher Cherry, Karlijn Hummelink, Kim Monkhorst, Xiaoshan M. Shao, Zineb Belcaid, Kristen A. Marrone, Joseph Murray, Kellie N. Smith, Benjamin Levy, Josephine Feliciano, Christine L. Hann, Vincent Lam, Drew M. Pardoll, Rachel Karchin, Tanguy Y. Seiwert, Julie R. Brahmer, Patrick M. Forde, Victor E. Velculescu, Valsamo Anagnostou

**Affiliations:** 1 The Sidney Kimmel Comprehensive Cancer Center, Johns Hopkins University School of Medicine, Baltimore, MD USA; 2 Netherlands Cancer Institute, Amsterdam, the Netherlands; 3 The Bloomberg-Kimmel Institute for Cancer Immunotherapy, Johns Hopkins University School of Medicine, Baltimore, MD USA; 4 Institute for Computational Medicine, Johns Hopkins University, Baltimore, MD USA

**Keywords:** Cancer genomics, Medical genomics, Immunotherapy

## Abstract

Tumor mutation burden is an imperfect proxy of tumor foreignness and has therefore failed to consistently demonstrate clinical utility in predicting responses in the context of immunotherapy. We evaluated mutations in regions of the genome that are unlikely to undergo loss in a pan-cancer analysis across 31 tumor types (*n* = 9,242) and eight immunotherapy-treated cohorts of patients with non-small-cell lung cancer, melanoma, mesothelioma, and head and neck cancer (*n* = 524). We discovered that mutations in single-copy regions and those present in multiple copies per cell constitute a persistent tumor mutation burden (pTMB) which is linked with therapeutic response to immune checkpoint blockade. Persistent mutations were retained in the context of tumor evolution under selective pressure of immunotherapy and tumors with a high pTMB content were characterized by a more inflamed tumor microenvironment. pTMB imposes an evolutionary bottleneck that cancer cells cannot overcome and may thus drive sustained immunologic tumor control in the context of immunotherapy.

## Abstract (web summary)

Genomic analyses in large cohorts of patients with cancer identify a new measure of tumor mutational burden, based on genomic regions that are unlikely to undergo loss, that is associated with therapeutic response to immunotherapy.

## Main

The current working hypothesis for tumor-intrinsic features that determine the magnitude of anti-tumor immune responses relies on the assumption that each mutation contributes equally to a composite measure of tumor foreignness, reflected in the number of sequence alterations per coding DNA sequence or tumor mutation burden (TMB). However, with the exception of mismatch repair-deficient tumors, TMB has failed to consistently demonstrate clinical utility in predicting responses to cancer immunotherapy. Efforts to separate subsets of alterations that may predominantly drive an effective anti-tumor immune response have yet to reveal a universal genomic predictive biomarker^1,2^. We have previously shown that heterozygous mutations and neoantigens can be selectively eliminated through chromosomal deletions and loss of heterozygosity (LOH) conferring acquired resistance to immune checkpoint blockade (ICB)^3^. In line with these findings, we have discovered that a higher number of sequence alterations contained in single-copy regions of the genome differentiate responding from nonresponding tumors in the context of immunotherapy^4^. Together, these findings suggested that mutations and associated neoantigens contained in regions of the genome present in a single copy per cancer cell are less likely to be eliminated by chromosomal loss under the selective pressure of therapy and therefore may mediate sustained neoantigen-driven immune responses and long-term clinical benefit^4^.

**Extended Data Fig. 1:**
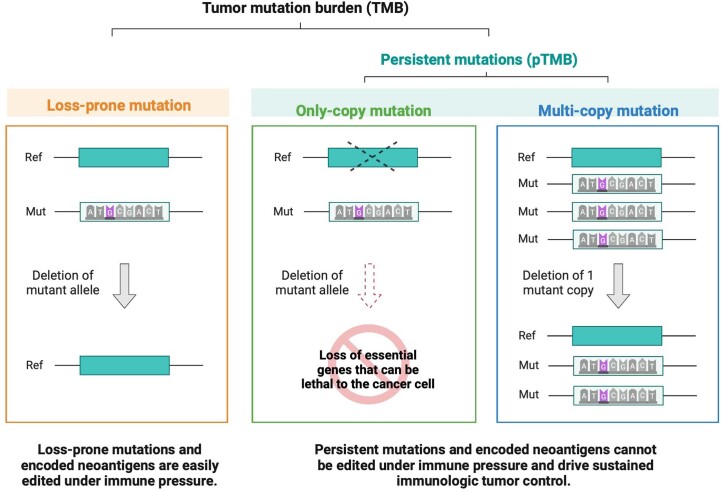
The persistent mutation hypothesis. We hypothesized that tumors with a higher frequency of sequence alterations in either haploid regions or in multiple copies would be intrinsically incapable of escaping immune recognition in the setting of immunotherapy, as these alterations would continuously render them visible to the immune system, resulting in sustained tumor rejection. These two distinct genomic mechanisms produce a common feature that we term “persistent mutations”, a key driver of continued immunologic tumor control.

To extend these findings beyond single-copy genomic regions, we hypothesized that tumors with a higher frequency of sequence alterations in either haploid regions or multiple copies would have a fitness disadvantage in the context of immunotherapy, as these alterations would continuously render them visible to the immune system, resulting in sustained immunologic tumor control. Deletions of single-copy alleles through chromosomal loss are typically not tolerated unless they are relatively small homozygous deletions^5^, as larger chromosomal deletions could contain essential genes in linkage with the mutation and thus be lethal. Similarly, mutation loss by chromosomal deletions^3^ is evolutionarily unlikely when mutations are contained in multiple copies. Therefore, these ‘persistent’ mutations (which we hereafter refer to as pTMB) may function as an intrinsic driver of tumor rejection in the tumor microenvironment (TME) (Extended Data Fig. 1).

## Results

**Fig. 1:**
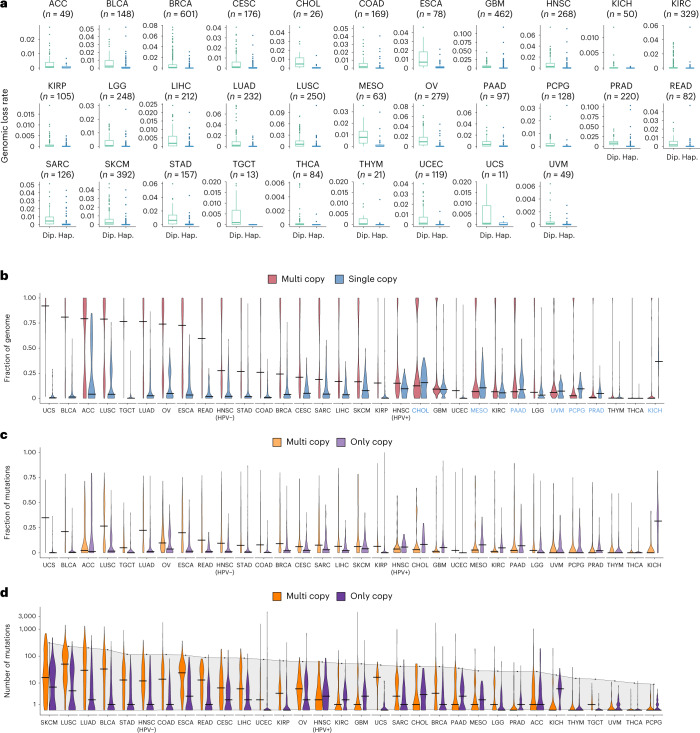
Pan-cancer distribution of persistent mutation load. **a**, The background rate of genomic loss was quantified in 31 tumor types from TCGA (*N* = 5,244). In all tumor types, the rate of loss was significantly higher in diploid (allele-specific copy numbers 1–1) versus haploid (single-copy; allele-specific copy numbers 1–0) regions of the genome (two-sided Mann–Whitney *U*-test, *P* < 0.05 for all tumor types except for UCS for which Mann–Whitney *P* = 0.067), supporting the notion that mutations that reside in haploid regions of the genome would less likely be lost. Box plots depict the median value and hinges correspond to the first and third quartiles. The whiskers extend from the corresponding hinge to the furthest value within 1.5 × interquartile range from the hinge. **b**, An analysis of somatic copy number aberrations in 9,991 tumors across 31 tumor types from TCGA identified the fraction of the genome with a single copy present (total copy number of 1) and with multiple copies of a parental haplotype. A differential enrichment pattern was noted, whereby cancers including UCS, BLCA, ACC, LUSC, LUAD, OV and SKCM had a higher fraction of the genome with multiple copies compared with CHOL, PAAD, MESO and KICH which showed a higher genome fraction in the only-copy state. Violin plots depict the distribution of genome fractions in each state, and the horizontal black segments indicate median values. Tumor types with predominance of single-copy genome fraction are marked in blue font. **c**, The prevalence of somatic mutations present in multiple copies per cell (multi-copy), and those present in haploid regions of the genome (only-copy), is depicted for 9,242 tumors, highlighting similar differential distributions per cancer type. **d**, The number of multi-copy and only-copy somatic mutations is shown against a background of the median TMB of the corresponding tumor type. Notably, the median TMB within a tumor type does not fully predict the abundance of multi-copy and only-copy mutations, and tumor types with very similar TMB may exhibit differences in multi-copy and only-copy mutation load (for instance, UCS versus GBM and SARC). Dip., diploid; Hap., haploid; HPV+, human papillomavirus positive; HPV−, human papillomavirus negative. Source data

**Fig. 2:**
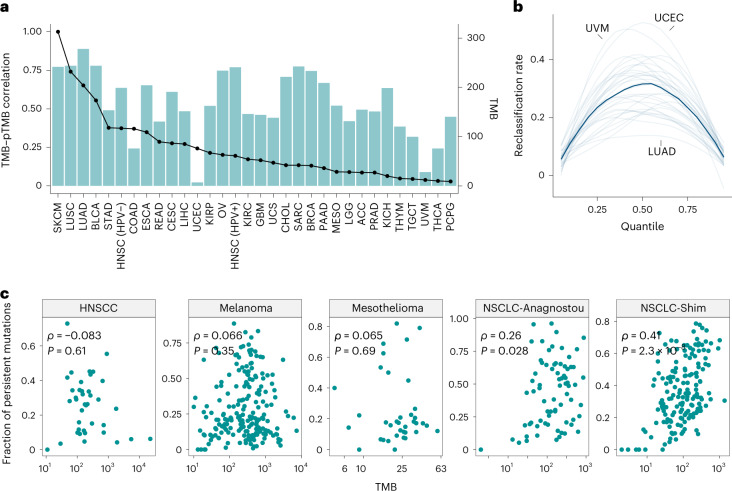
Evaluation of the association between persistent mutation content and TMB. **a**, Analysis of 9,242 tumors across 31 tumor types revealed a large variation in the correlations between TMB and pTMB (blue bars), which is not entirely explained by TMB alone (median TMB values within each tumor type are shown in solid trace). **b**, The tumor reclassification rate was calculated by applying a given quantile of TMB and pTMB and is shown for 31 tumor types within the TCGA cohort (semi-transparent traces), with the median reclassification rate depicted in a solid line. In tumors such as UVM and UCEC, up to 40% of the samples could be differentially classified as pTMB-high versus pTMB-low using persistent mutations rather than the overall TMB value. **c**, The fraction of persistent mutations exhibited a variable degree of correlation with TMB in five ICB-treated cohorts (*n* = 524) across four tumor types. In HNSCC (*n* = 39), melanoma (*n* = 202) and mesothelioma (*n* = 40), no significant correlation was observed (HNSCC: Spearman *ρ* = -0.083 and *P* = 0.61; melanoma: *ρ* = 0.066 and *P* = 0.35; mesothelioma: *ρ* = 0.065 and *P* = 0.69; two-sided *P* values were calculated assuming asymptotic *t* approximation), while a weak/moderate correlation was observed in NSCLC (NSCLC-Anagnostou, *n* = 74, Spearman *ρ* = 0.26, *P* = 0.028; and NSCLC-Shim, *n* = 169, Spearman *ρ* = 0.41, *P* = 2.3 × 10^−8^). Spearman’s rank correlation coefficients and corresponding *P* values are shown in inserts in panel **c**.

**Extended Data Fig. 2:**
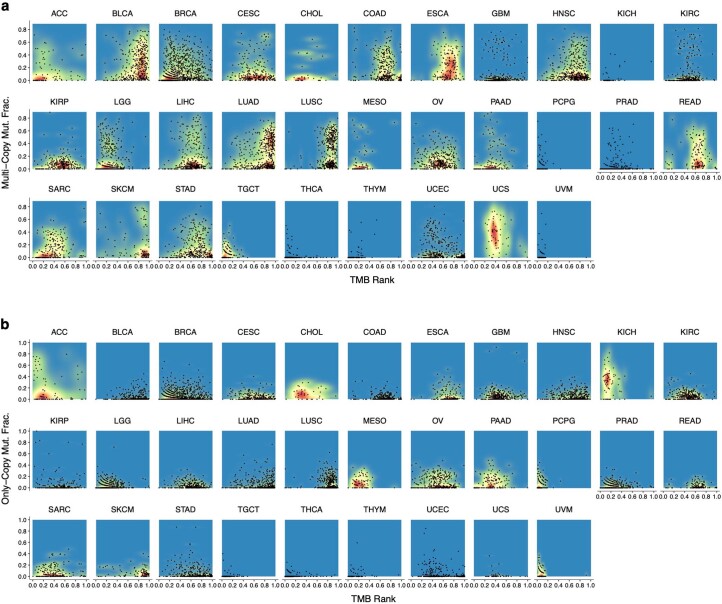
Interaction of TMB and fraction of mutations in single- and multi-copy regions across 31 cancer types. A combined analysis of TMB ranks in relation to the fraction of mutations in multi-copy (a) and only-copy (b) regions of the genome revealed a complex landscape, where a wide range of multi-copy or single-copy mutation fraction was observed at any given TMB value in 9,256 tumors across 31 cancer types from TCGA. These findings highlight tumors with a low or intermediate TMB that at the same time harbor a large fraction of mutations in multi-copy or single-copy states, therefore suggesting the independent nature of these features. A weak correlation was noted between TMB rank and fraction of mutations in multi-copy regions of the genome for BLCA (Pearson’s R = 0.26), KICH (Pearson’s R = 0.45), LUAD (Pearson’s R = 0.34), LUSC (Pearson’s R = 0.21), SARC (Pearson’s R = 0.24) while these features were weakly anti-correlated in COAD and UCEC (Pearson’s R = -0.23 and -0.35 respectively). TMB was weakly anti-correlated with the fraction of mutations in haploid regions for COAD, STAD and UCEC (Pearson’s R = -0.22, −0.25 and −0.25 respectively). The heatmaps indicate the distribution of tumors within a tumor type, and each black dot represents an analyzed tumor sample. Source data

**Extended Data Fig. 3:**
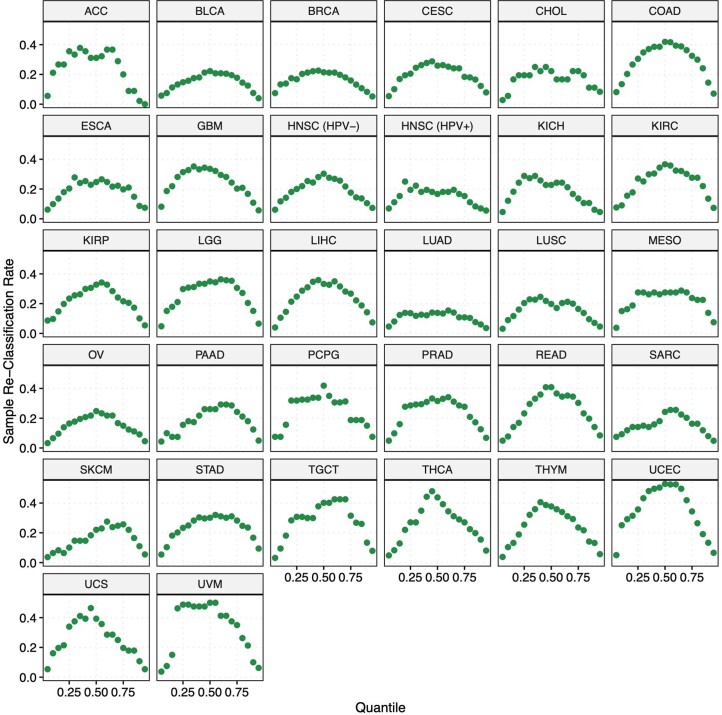
Tumor re-classification by pTMB vs TMB. A series of quantile values going from 5% to 95% in 5% increments were used to extract samples with high and low values for each predictor variable in each tumor type. For each quantile value, the re-classification rate per samples within a cancer type group. By definition, sample re-classification rate starts close to 0 at the lower end of quantile thresholds (where almost all samples are labeled as high regardless of the metric used), and returns to 0 at the higher end of quantile thresholds (where the great majority of samples are labeled as low irrespective of the metric used). In the intermediate range, re-classification rates as high as 50% were observed.

To investigate these hypotheses in a pan-cancer manner, we first evaluated the rate of loss in regions of the genome with a single copy per cell (haploid) versus euploid regions (two copies per cell) using copy number profiles of 5,244 tumors across 31 tumor types from The Cancer Genome Atlas (TCGA) (Supplementary Table 1). These analyses revealed that the rate of loss in haploid regions was consistently lower than that in euploid regions (Fig. 1a), supporting the notion that mutations contained in these regions would be difficult to eliminate. We then examined the frequency of haploid and polyploid regions across the genome and quantified the fraction of the genome in single-copy versus multi-copy states (*N* = 9,991; Fig. 1b and Supplementary Table 2). Some tumor types, including endometrial carcinosarcomas, bladder cancers, adrenocortical carcinomas, lung squamous carcinomas, lung adenocarcinomas, ovarian cancers and cutaneous melanomas, were enriched for genomic regions in the multi-copy state, while cholangiocarcinomas, pancreatic adenocarcinomas, mesotheliomas and kidney chromophobe tumors showed a higher genome fraction in the single-copy state (Fig. 1b). Integration of sequence alterations in only-copy and multi-copy states revealed a cancer lineage-dependent distribution of persistent mutations (Fig. 1c,d and Supplementary Table 2). Next, we characterized the distribution of persistent mutation load in the background of the overall TMB within each tumor type, and found that TMB does not fully explain the abundance of multi-copy and only-copy mutations, as tumor types with similar TMB exhibited differences in multi-copy and only-copy mutation content (*N* = 9,242; Fig. 1d and Supplementary Table 2). Notably, a wide range of prevalence of mutations in only-copy or multi-copy states was observed across the range of overall TMB, suggesting that persistent mutations provide a measure of alterations that is distinct from TMB (Extended Data Fig. 2). We further evaluated the degree of correlation between TMB and pTMB and found a substantial degree of variation in their association across the 31 tumor types analyzed (Spearman *ρ*: median 0.49, range: 0.02–0.89; Fig. 2a and Supplementary Table 3). Similar patterns were observed when multi-copy (Spearman *ρ* median: 0.42, range: 0.02–0.76) and single-copy mutations (Spearman *ρ* median: 0.21, range: −0.12 to 0.48) were considered separately. To understand the potential reclassification of tumors based on pTMB, we employed a series of quantile values ranging from 5% to 95% to define high/low groups for TMB and pTMB (Methods). These analyses revealed reclassification rates as high as 53% (range: 15–53%), with a median reclassification rate of 33% across all tumor types (Fig. 2b and Extended Data Fig. 3).

Next, we explored the relationship between persistent mutation content and TMB in seven published ICB cohorts across three tumor types (*n* = 485; melanoma^6–9^, non-small-cell lung cancer (NSCLC)^2,10^ and mesothelioma^11^) and a new cohort of patients with HPV-negative head and neck cancer (head and neck squamous cell carcinoma (HNSCC)) who received ICB (*n* = 39; Supplementary Tables 4–8). Similar to the TCGA analyses, we did not detect a significant enrichment for a higher persistent mutation fraction in tumors harboring a higher TMB in the HNSCC (Spearman *ρ* = −0.083, *P* = 0.61), melanoma (Spearman *ρ* = 0.066, *P* = 0.35) and mesothelioma cohorts (Spearman *ρ* = 0.065, *P* = 0.69), while a weak correlation between TMB and persistent mutation fraction was observed in the NSCLC cohorts (NSCLC-Anagnostou: Spearman *ρ* = 0.26, *P* = 0.03; NSCLC-Shim: Spearman *ρ* = 0.41, *P* = 2.3 × 10^−8^; Fig. 2c and Supplementary Table 9). Collectively, these findings further support the notion that pTMB is distinct from TMB and that tumors are differentially ranked by their pTMB content in a cancer lineage-dependent manner.

**Extended Data Fig. 4:**
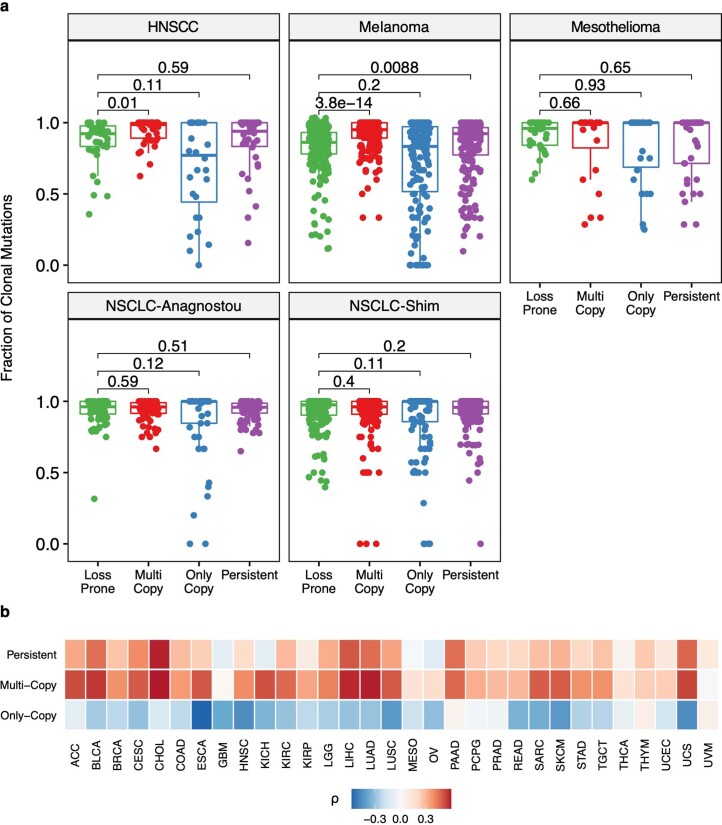
Clonal architecture of persistent mutations. **(a)** We computed the fraction of clonal mutations within loss-prone, multi-copy, only-copy, and persistent mutations sets. In the HNSCC (n = 39) and melanoma cohorts (n = 208), multi-copy mutations were more clonal compared to loss-prone mutations (HNSCC; p = 0.01, melanoma; p = 3.8e-14), but no such difference was present in the mesothelioma (n = 40, p = 0.66) and NSCLC cohorts (NSCLC-Anagnostou; n = 75 and p = 0.59, NSCLC-Shim; n = 169 and p = 0.40). Only-copy mutations had similar clonal fractions as loss-prone mutations (p > 0.11) in all five cohorts. When multi-copy and only-copy mutations were considered together, we did not identify a significant difference in clonal fractions between persistent and loss-prone mutations in the HNSCC (p = 0.59), mesothelioma (p = 0.65), and NSCLC cohorts (NSCLC-Anagnostou; p = 0.51, NSCLC-Shim; p = 0.20). Persistent mutations tended to be more clonal in the melanoma cohort (p = 8.80e-03). Box plots depict the median value and hinges correspond to the first and third quartiles. The whiskers extend from the corresponding hinge to the furthest value within 1.5 * the interquartile range from the hinge. Two-sided Mann-Whitney U-test was used to compare values in across mutation classes. **(b)** A weak to moderate degree of correlation was observed between the fraction of clonal mutations and the number of persistent mutations (Spearman ρ range: -0.11 - 0.59) or multi-copy mutations (Spearman ρ range: 0.01 - 0.60) across the 31 TCGA tumor types analyzed. Notably, the number of only-copy mutations was anti-correlated with the fraction of clonal mutations in the TCGA dataset (Spearman ρ range: -0.57, 0.07). Spearman’s rank correlation coefficients are depicted in the heatmap. Source data

We theorized that the impact of pTMB would be exemplified in the context of ICB treatment, where inherent anti-tumor immune responses would be enhanced and sustained in the presence of continued persistent mutation-associated neoantigen (pMANA) stimulation. As our analyses pointed towards subsets of mutations within TMB that may carry differential weights in demarcating tumor foreignness, we evaluated persistent mutations in comparison with mutations that are more likely to be lost in the context of tumor evolution. We refer to the latter as ‘loss-prone’ mutations and these account for the majority of coding alterations that constitute a tumor’s TMB. We next asked the question of whether there are differential clonal compositions between the persistent and loss-prone mutation subsets. In the HNSCC, mesothelioma and NSCLC cohorts, we did not detect a difference in the fraction of clonal alterations between persistent mutations and loss-prone mutations, while in the melanoma cohort, persistent mutations tended to be more clonal (Extended Data Fig. 4). When multi-copy mutations were considered separately, higher cellular fractions were noted for the multi-copy subset compared with loss-prone mutations in melanoma and HNSCC, in line with the notion that multi-copy mutations may be acquired before somatic copy number gains. To further study the clonal architecture of persistent mutations, we evaluated the correlation between pTMB and the fraction of clonal mutations in 31 TCGA tumor types and in the ICB cohorts. In the TCGA dataset, we observed a wide range of correlations between pTMB and fraction of clonal mutations (Spearman *ρ* range: −0.11 to 0.59; Extended Data Fig. 4 and Supplementary Table 3). We found a moderate degree of anti-correlation between clonal mutation fraction and the number of only-copy mutations in most tumor types (Spearman *ρ* range: −0.57 to 0.07), while a moderate degree of correlation was detected between the fraction of clonal mutations and multi-copy mutations (Spearman *ρ* range: 0.01–0.60; Extended Data Fig. 4). In the ICB cohorts, we did not detect strong correlations between tumor clonal heterogeneity and persistent mutations (Spearman *ρ* range: −0.25 to 0.33) (Supplementary Fig. 1 and Supplementary Table 9). These findings suggest that persistent mutations are encountered at the full spectrum of tumor clonal heterogeneity.

**Extended Data Fig. 5:**
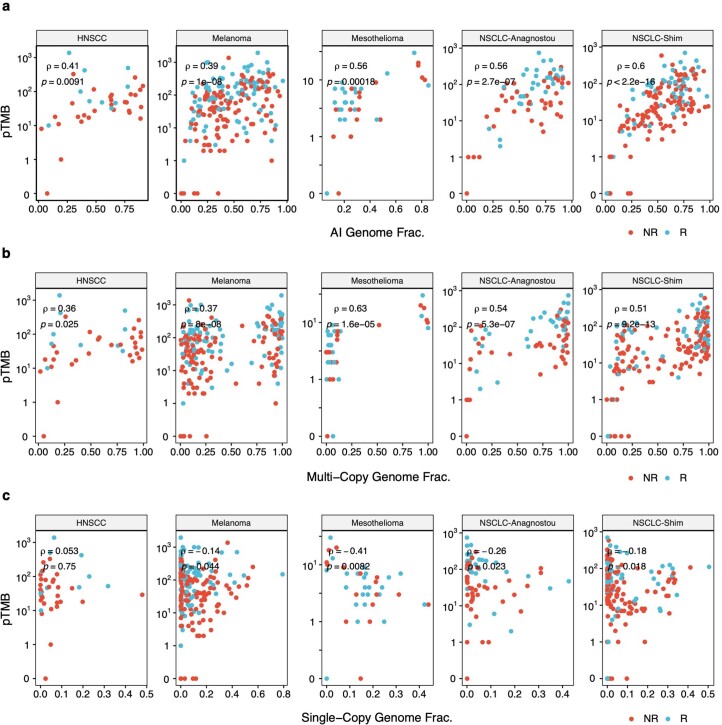
Correlations between persistent mutations and tumor aneuploidy. **(a)** A moderate degree of correlation was observed between pTMB and the fraction of genome with allelic imbalance in the five ICB cohorts analyzed (HNSCC Spearman ρ = 0.41, p = 9e-3; Melanoma ρ = 0.39, p = 1e-8; Mesothelioma ρ = 0.56, p = 1.8e-4; NSCLC-Anagnostou ρ = 0.56, p = 2.7e-7; NSCLC-Shim ρ = 0.60, p = 2.2e-16) indicating that tumors with higher levels of aneuploidy tend to have higher pTMB. **(b)** Similar levels of correlation were observed between pTMB and the fraction of genome with multiple (>2) copies (HNSCC Spearman ρ = 0.36, p = 0.03; Melanoma ρ = 0.37, p = 8e-8; Mesothelioma ρ = 0.63, p = 1.6e-5; NSCLC-Anagnostou ρ = 0.54, p = 5.3e-7; NSCLC-Shim ρ = 0.51, p = 9.2e-13). **(c)** pTMB was weakly anti-correlated with the fraction of genome at single copy number (total CN = 1) in the melanoma (ρ = -0.14, p = 0.04), mesothelioma (ρ = -0.41, p = 8e-3), and NSCLC ICB cohorts (NSCLC-Anagnostou ρ = -0.26, p = 0.02; NSCLC-Shim ρ = -0.18, p = 0.02). Spearman’s rank correlation coefficients are shown, each tumor sample represents a point and points are color coded based on tumor response on ICB. Two-sided p-values are calculated using asymptotic t approximation.

Conceptually, persistent mutations, which by definition reside in aneuploid regions of the genome, are integrally linked with tumor aneuploidy and, next, we assessed the relationship between persistent mutations and fraction of the genome with allelic imbalance. In the ICB cohorts, a moderate degree of correlation was observed between tumor aneuploidy and pTMB (Spearman *ρ* range: 0.39–0.60; Extended Data Fig. 5). In parallel, we evaluated tumors for whole-genome doubling (WGD) events, which would enable the acquisition of additional mutant copies of the mutations present before the doubling event, and may therefore be a critical contributor to pTMB. Indeed, in all ICB cohorts analyzed, tumors with WGD also harbored a higher number of multi-copy mutations (HNSCC, Mann–Whitney *P* = 1.6 × 10^−5^; melanoma, *P* = 3.14 × 10^−14^; mesothelioma, *P* = 8.24 × 10^−6^; NSCLC-Anagnostou, *P* = 6.82 × 10^−9^; NSCLC-Shim, *P* = 9.3 × 10^−17^; Supplementary Fig. 2). Notably, tumors that have undergone WGD are by definition expected to have a very small fraction of the genome at total copy number of 1, and, consistent with this notion, we observed a much lower prevalence of only-copy mutations in genomes with WGD.

**Extended Data Fig. 6:**
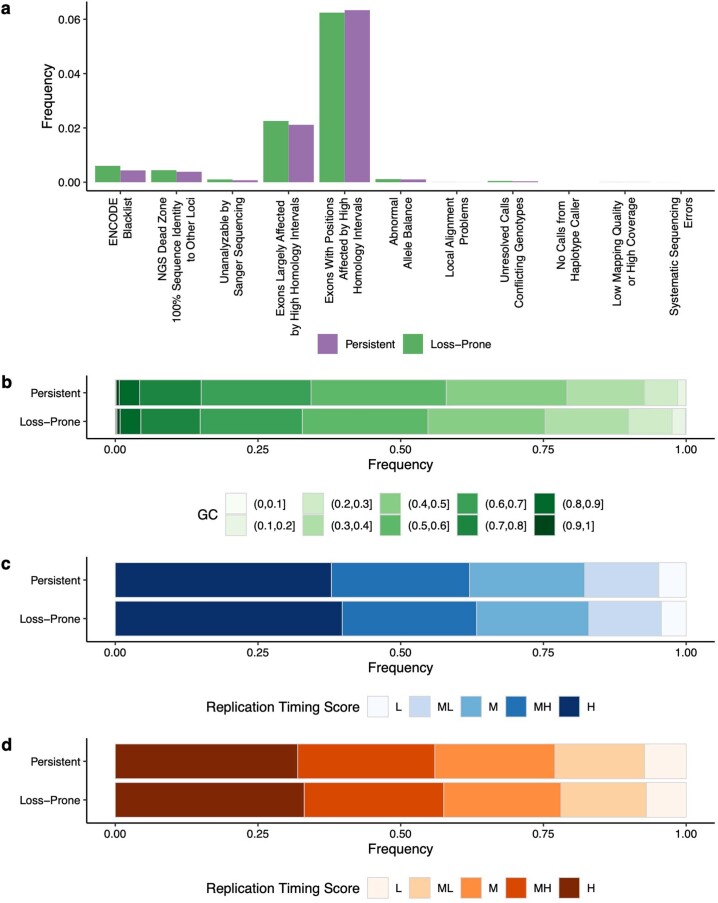
Assessment of genomic characteristics of loci harboring persistent vs loss-prone mutations in TCGA tumors. **(a)** Genomic regions that are susceptible to limitations of NGS analysis, such as uncertain alignments or variant calls, were infrequent in the somatic mutation call set analyzed (MC3), and had similar representation in the persistent and loss-prone mutation categories (n = 9,242). **(b)** Similarly, the difference in GC composition of the immediate bases (9-mers) surrounding persistent and loss-prone mutations was negligible (n = 9,242; Cohen’s d = 0.08, persistent mean = 0.54, loss-prone mean = 0.52). Persistent and loss-prone mutations were found to have similar replication timing in **(c)** melanoma (TCGA-SKCM, n = 109, Cohen’s d = -0.035) and **(d)** NSCLC (combined set of TCGA-LUAD and TCGA-LUSC, n = 982, Cohen’s d = -0.032). Stacked bar plots depict the frequency of mutations in the five quantiles of replication timing. H, MH, M, ML, and L indicate mutations in the highest to lowest quintiles of replication timing in order; as an example mutations in earliest replicating regions are marked as H.

We investigated potential bias related to different timing of acquisition of persistent mutations, background mutation rates and accuracy of mutation calls in these loci and evaluated the distribution of sequence properties such as GC content and replication timing as well as mutation call quality in persistent versus loss-prone mutations in 9,242 tumors from TCGA (Extended Data Fig. 6). We found a similar GC composition surrounding loci with persistent and loss-prone mutations (Cohen’s *d* = 0.08, persistent mean = 0.54, loss-prone mean = 0.52). We then performed a cancer lineage-specific evaluation of replication timing in the melanoma and NSCLC subsets and found a similar distribution in persistent and loss-prone mutations (melanoma Cohen’s *d* = −0.035, NSCLC Cohen’s *d* = −0.032). Furthermore, we quantified the fraction of mutations in each category that could theoretically be affected by limitations of next-generation sequencing (NGS) analysis^12,13^ and found similar distributions in persistent and loss-prone mutations (Extended Data Fig. 6). These findings suggest that persistent mutation calling is not confounded by background mutation rates, replication timing or technical artifacts.

**Extended Data Fig. 7:**
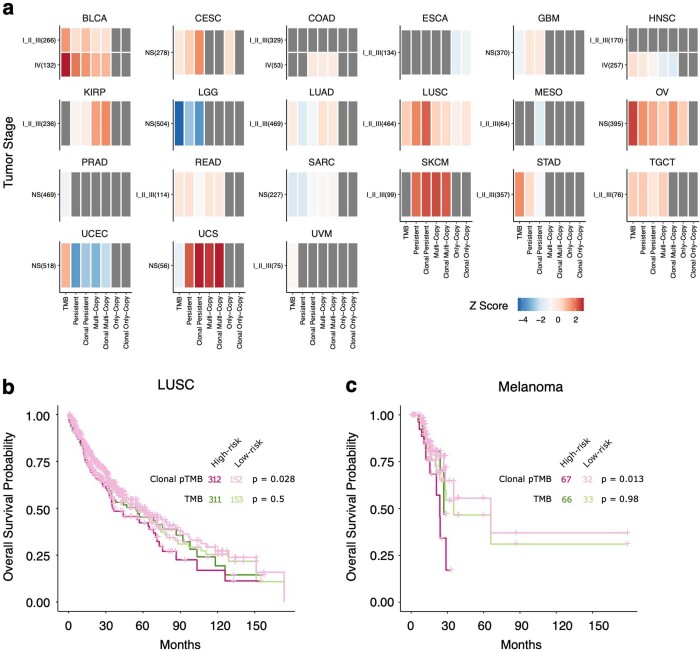
Context dependence of the association between pTMB and overall survival. (a) The association between persistent mutations and TMB with overall survival was assessed for 8,925 individuals across 31 cohorts in TCGA. For each tumor type and stage combination, a Cox proportional-hazards (CoxPH) model predicting the overall survival is built for each of seven features (TMB, persistent mutations-pTMB, clonal pTMB, multi-copy mutations, clonal multi-copy mutations, only-copy mutations, clonal only-copy mutations; Continuous CoxPH model). In 21 tumor types shown, an increase in at least one of the seven features listed was associated with longer overall survival and a second CoxPH model was used to assess the difference in overall survival between tumors in the top third and bottom two thirds of predicted risk (Categorical CoxPH model). Heatmap cells depict the Z-score of the model coefficient from categorical CoxPH model. Grey indicates cases where an increase in feature value is associated with shorter survival. A significant association of pTMB with prolonged overall survival was noted for lung squamous cell carcinoma, melanoma and UCS. (b) Patients with early stage (I, II, and III) squamous lung cancer (n = 464) harboring a high pTMB (low risk) had a significantly longer overall survival compared to patients in the low pTMB (high risk) group especially when clonal pTMB was considered (pTMB: 56.27 vs 43.86 months, log-rank p = 0.085; clonal pTMB: 60.48 vs 35.32 months, log-rank p = 0.028), while no difference was found between high vs. low TMB risk groups (TMB: 55.16 vs 54.37 months, log-rank p = 0.50). (c) Similarly, for patients with early stage melanoma (n = 99), tumors with higher pTMB (low risk) had a significantly longer overall survival compared to those with low pTMB especially when clonal pTMB was considered (pTMB: 65.83 vs 23.69 months, log-rank p = 0.036; clonal pTMB: 65.83 vs 23.69 months, log-rank p = 0.013); while no such difference was observed for TMB-high vs low tumors (35.15 vs 26.97 months, log-rank p = 0.98).

We then evaluated whether a higher pTMB was linked with clinical outcome in patients with previously untreated tumors from TCGA (Methods). Our analyses showed that the association between pTMB and clinical outcome was context-dependent, whereby a significant association with prolonged overall survival was noted for lung squamous cell carcinoma (pTMB: 56.27 versus 43.86 months, log-rank *P* = 0.085; clonal pTMB: 60.48 versus 35.32 months, log-rank *P* = 0.028), melanoma (pTMB: 65.83 versus 23.69 months, log-rank *P* = 0.036; clonal pTMB: 65.83 versus 23.69 months, log-rank *P* = 0.013) and uterine carcinosarcoma (pTMB: 27.53 versus 17.15 months, log-rank *P* = 0.021; clonal pTMB: 50.13 versus 14.68 months, log-rank *P* = 2.66 × 10^−3^), but not for any other cancer type studied (Extended Data Fig. 7 and Supplementary Table 10). TMB was more weakly associated with overall survival in the lung squamous cell carcinoma (log-rank *P* = 0.50), melanoma (log-rank *P* = 0.98) and uterine carcinosarcoma (log-rank *P* = 0.17) sets.

**Fig. 3:**
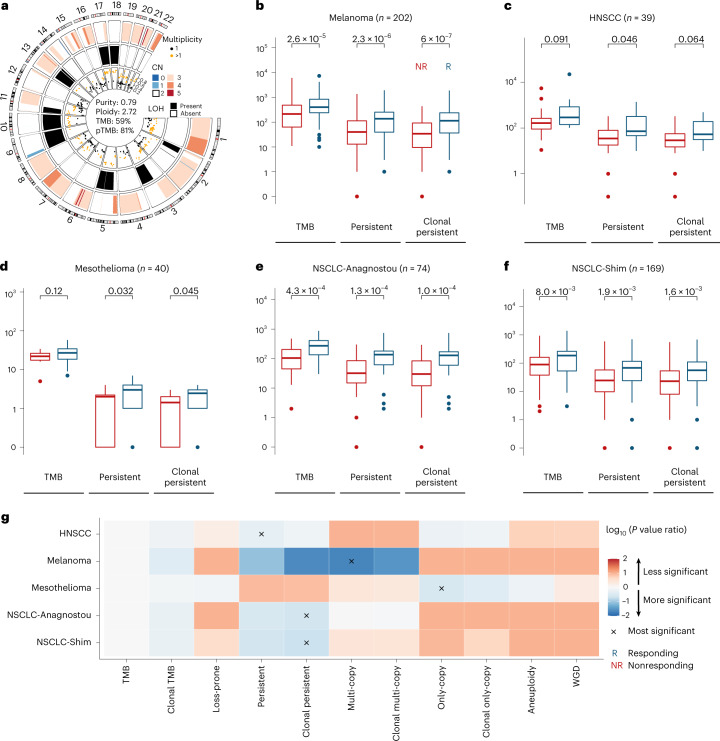
pTMB is linked with therapeutic response with ICB. **a**, A representative example of a responding tumor harboring an intermediate TMB (59% quantile) but high pTMB (81% quantile). The outer and middle ring depict segmental copy number and LOH, respectively. The genomic coordinates of mutations and their mutant allele fraction are shown in the inner ring, colored by their estimated multiplicity. **b**, In melanoma (*n* = 202; NR = 115, R = 87), pTMB distinguished responding from nonresponding tumors (MW *P* = 2.3 × 10^−6^ for pTMB, *P* = 6.0 × 10^−7^ for clonal pTMB) more optimally compared with TMB (MW *P* = 2.6 × 10^−5^). Box plots depict the median value and hinges correspond to the first and third quartiles. The whiskers extend from the corresponding hinge to the furthest value within 1.5 × interquartile range from the hinge. **c**, In HPV-negative HNSCC, we found a greater difference in pTMB of responding versus nonresponding tumors compared with TMB (*n* = 39; NR = 29, R = 10; MW *P* = 0.046 for pTMB, *P* = 0.064 for clonal pTMB, *P* = 0.091 for TMB). **d**, For mesotheliomas, pTMB outperformed TMB in prediction of response to chemo-immunotherapy (*n* = 40; NR = 16, R = 24; MW *P* = 0.032 for pTMB, *P* = 0.045 for clonal pTMB, non-significant for TMB). **e**,**f**, In NSCLC, a higher pTMB differentiated responding from nonresponding tumors (NSCLC-Anagnostou: *n* = 74; NR = 41, R = 33; MW *P* = 1.3 × 10^−4^ for pTMB, *P* = 1.0 × 10^−4^ for clonal pTMB, *P* = 4.3 × 10^−4^ for TMB; NSCLC-Shim: *n* = 169; NR = 49, R = 120; MW *P* = 1.9 × 10^−3^ for pTMB, *P* = 1.6 × 10^−3^ for clonal pTMB, *P* = 8.0 × 10^−3^ for TMB). **g**, The significance of association of eight mutation-based and two copy number-based features with therapeutic response was compared with that of TMB (log_10_ of feature to TMB *P* value ratios visualized). For each cohort, the feature with the most significant association with therapeutic response is marked with an ‘x’ mark. For all ICB cohorts, pTMB outperformed TMB in predicting ICB response. Notably, the best-performing feature in NSCLC was clonal pTMB (MW *P* = 1.03 × 10^−4^, *P* = 1.60 × 10^−3^ for NSCLC-Anagnostou and NSCLC-Shim, respectively), while in melanoma the number of multi-copy persistent mutations (MW *P* = 5.42 × 10^−7^) and in mesothelioma the number of only-copy persistent mutations (MW *P* = 3.15 × 10^−2^) better distinguished between responding and nonresponding tumors. pTMB outperformed loss-prone mutation load in distinguishing responding from nonresponding tumors (HNSCC: MW *P* = 0.16 versus *P* = 0.05; melanoma: *P* = 1.92 × 10^3^ versus *P* = 2.25 × 10^−6^; mesothelioma: *P* = 0.09 versus *P* = 0.03; NSCLC-Anagnostou: *P* = 1.03 × 10^−2^ versus *P* = 1.26 × 10^−4^; NSCLC-Shim: *P* = 3.20 × 10^−2^ versus *P* = 1.87 × 10^−3^). Tumor aneuploidy or WGD alone failed to predict ICB response. *P* values are two-sided. CN, copy number; MW, Mann–Whitney; NR, nonresponding; R, responding. Source data

Importantly, we hypothesized that tumors with a high persistent mutation content would be the most ‘visible’ to the immune system and would therefore regress in the context of immunotherapy, a phenomenon that would be reflected in sustained clinical responses to therapy. To this end, we evaluated the potential of pTMB, multi-copy and only-copy mutations in predicting ICB response in 524 patients with melanoma, NSCLC, mesothelioma and HNSCC (Methods and Supplementary Table 9). We discovered that tumors with a high pTMB attained higher rates of therapeutic response with ICB, while TMB alone or the number of loss-prone mutations less optimally distinguished responding from nonresponding tumors (Fig. 3 and Supplementary Table 11). As a representative example that illustrates the difference between pTMB and TMB, patient 44 with metastatic melanoma harboring a pTMB in the 81% quantile but in the 59% quantile by TMB attained a prolonged progression-free survival on ICB (Fig. 3a). High pTMB more accurately differentiated responding from nonresponding tumors in the melanoma cohort (*n* = 202, Mann–Whitney *U*-test *P* = 2.3 × 10^−6^, *P* = 6.0 × 10^−7^, *P* = 1.92 × 10^−3^ and *P* = 2.6 × 10^−5^ for pTMB, clonal pTMB, loss-prone mutation load and TMB, respectively; Fig. 3b). Similarly, in the HNSCC ICB cohort, pTMB was associated with therapeutic response (*n* = 39, Mann–Whitney *U*-test *P* = 0.05, *P* = 0.06, *P* = 0.16 and *P* = 0.09 for pTMB, clonal pTMB, loss-prone mutations and TMB, respectively; Fig. 3c). In the mesothelioma cohort, pTMB outperformed TMB in predicting response to durvalumab plus platinum-pemetrexed chemotherapy (*n* = 40, Mann–Whitney *U*-test *P* = 0.03, *P* = 0.05, *P* = 0.09 and *P* = 0.12 for pTMB, clonal pTMB, loss-prone mutations and TMB, respectively; Fig. 3d). A higher pTMB differentiated responding from nonresponding NSCLC (NSCLC-Anagnostou, *n* = 74, Mann–Whitney *U*-test *P* = 1.3 × 10^−4^, *P* = 1.0 × 10^−4^, *P* = 0.01 and *P* = 4.3 × 10^−4^ for pTMB, clonal pTMB, loss-prone mutations and TMB, respectively; NSCLC-Shim, *n* = 169, *P* = 1.9 × 10^−3^, *P* = 1.6 × 10^−3^, *P* = 0.03 and *P* = 8.0 × 10^−3^ for pTMB, clonal pTMB, loss-prone mutations and TMB, respectively; Fig. 3e,f).

Next, we evaluated the effect size of persistent mutations, loss-prone mutations and TMB on clinical outcome (Supplementary Table 11). In the melanoma, HNSCC and mesothelioma cohorts, the effect size for pTMB was larger than TMB or loss-prone mutations (HNSCC: pTMB Cohen’s *d* = −0.96, TMB *d* = −0.64, loss-prone *d* = −0.61; melanoma: pTMB *d* = −0.57, TMB *d* = −0.44, loss-prone *d* = −0.35; mesothelioma: pTMB *d* = −0.74, TMB *d* = −0.51, loss-prone *d* = −0.58). In the NSCLC cohorts, the effect size for pTMB, while very close to that of TMB, exceeded the effect size of loss-prone mutations (NSCLC-Anagnostou: pTMB *d* = −0.89, TMB *d* = −0.93, loss-prone *d* = −0.58; NSCLC-Shim: pTMB *d* = −0.53, TMB *d* = −0.54, loss-prone *d* = −0.44). These findings suggest that the power of TMB to distinguish between responding and nonresponding tumors in the context of ICB is largely driven by their persistent mutation content. Notably, in the NSCLC cohort, clonal pTMB more optimally distinguished responding from nonresponding tumors (Mann–Whitney *U*-test *P* = 1.03 × 10^−4^ and *P* = 1.60 × 10^−3^ for NSCLC-Anagnostou and NSCLC-Shim, respectively). In the melanoma cohort, the number of multi-copy mutations was tightly correlated with therapeutic response (Mann–Whitney *U*-test *P* = 5.42 × 10^−7^), while in the mesothelioma cohort, the number of only-copy mutations better distinguished responding and nonresponding tumors (Mann–Whitney *U*-test *P* = 3.15 × 10^−2^; Fig. 3g). Importantly, pTMB outperformed loss-prone mutation content in all ICB cohorts, despite the latter representing a larger fraction of the overall TMB (Mann–Whitney *U*-test *P* = 1.92 × 10^−3^ versus 2.25 × 10^−6^, *P* = 0.16 versus 0.05, *P* = 0.09 versus 0.03, 1.03 × 10^−2^ versus 1.26 × 10^−4^, *P* = 3.20 × 10^−2^ versus 1.87 × 10^−3^ for loss-prone versus pTMB in melanoma, HNSCC, mesothelioma, NSCLC-Anagnostou and NSCLC-Shim, respectively; Fig. 3g). We next asked the question of whether the tumors differentially classified by pTMB compared with TMB have different responses to ICB. To this end, we evaluated the number of cases with differential pTMB/TMB classification and compared the therapeutic response rates between pTMB-low/TMB-high and pTMB-high/TMB-low tumors. In the melanoma cohort, 23 tumors fell in the TMB-high/pTMB-low category and 23 tumors fell in the TMB-low/pTMB-high category. We found a higher frequency of responding tumors in the pTMB-high/TMB-low category compared with the pTMB-low/TMB-high group (Fisher’s exact *P* = 0.04, pTMB-high/TMB-low group: 16 responders, 7 nonresponders; pTMB-low/TMB-high group: 8 responders, 15 nonresponders). These findings tie into the reclassification analyses from the TCGA cohorts, and together support a differential classification of tumors based on their persistent mutation content, which is reflective of improved outcomes in the ICB setting.

**Extended Data Fig. 8:**
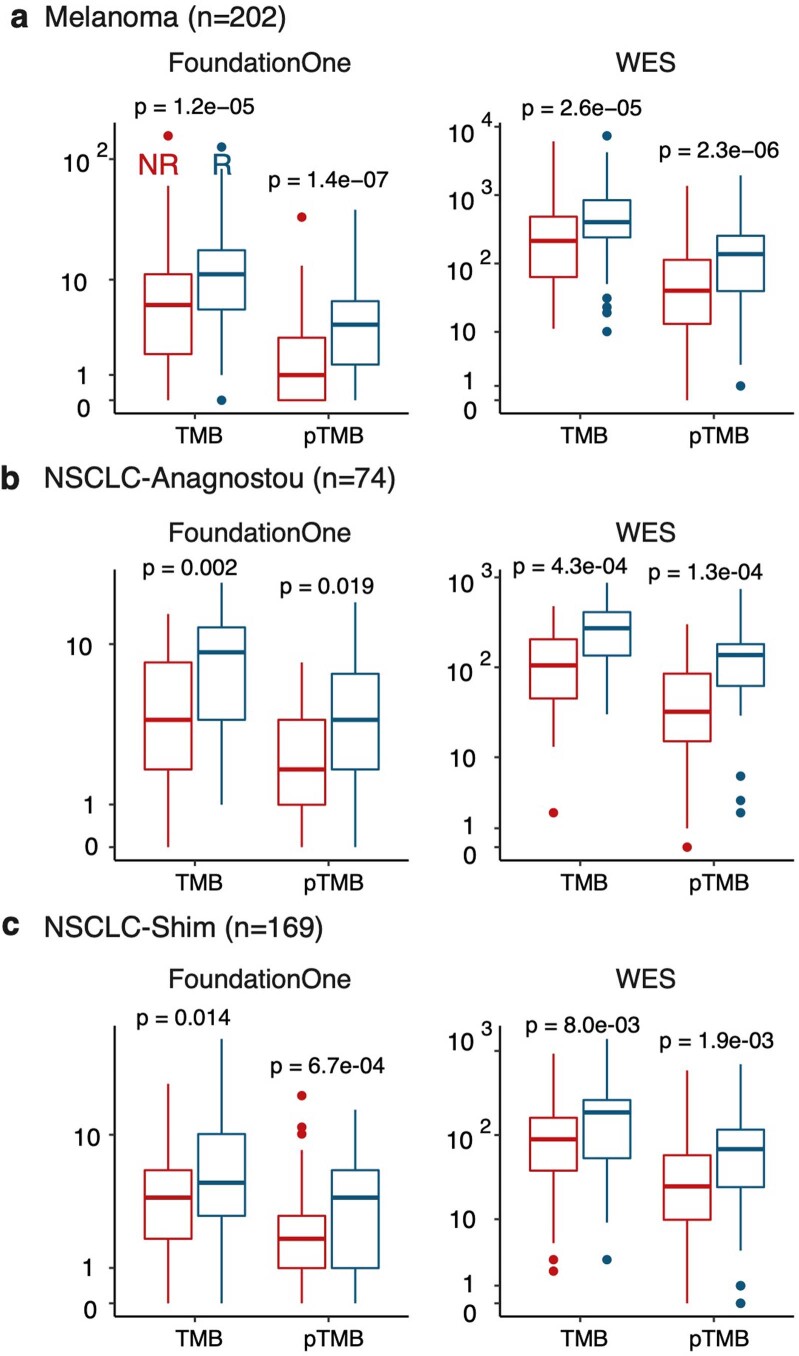
Evaluation of the association between pTMB computed by gene panel NGS and response to immune checkpoint blockade. TMB and pTMB estimates were derived by restricting analysis to the targeted intervals of a commonly used commercial 309 gene panel (FoundationOne-CDx) through an *in silico* simulation and compared between response groups. pTMB better distinguished between responding and non-responding tumors in melanoma (**a**; pTMB MW p = 1.3e-07 vs TMB MW p = 1.2e-05, NR n = 115, R n = 87; two-sided test) and the NSCLC-Shim cohort (**c**; pTMB MW p = 6.7e-04 vs TMB MW p = 0.014, NR n = 120, R n = 49; two-sided test). Comparison of TMB and pTMB between response groups using original estimates from whole exome sequence (WES) are included in each row for reference. pTMB and TMB had similar performance in the NSCLC-Anagnostou cohort (**b**), which was likely driven by the smaller size of this cohort (NR n = 41, R n = 33). Box plots depict the median value and hinges correspond to the first and third quartiles. The whiskers extend from the corresponding hinge to the furthest value within 1.5 * the interquartile range from the hinge. Source data

To further explore the immediate clinical utility of pTMB, we evaluated the feasibility of estimating pTMB from gene panel-targeted NGS, which is widely used in clinical cancer care. Using the genomic intervals from a widely adopted targeted NGS gene panel (309 genes; Methods), we performed in silico simulations utilizing whole exome sequence data from the melanoma and NSCLC ICB cohorts and computed TMB and pTMB in each tumor as captured by the region of interest of the targeted NGS panel. pTMB more accurately distinguished responding from nonresponding tumors (melanoma, *n* = 202, *P* = 1.37 × 10^−7^ for pTMB and *P* = 1.22 × 10^−5^ for TMB; NSCLC-Shim, *n* = 169, *P* = 6.7 × 10^−4^ for pTMB and *P* = 0.014 for TMB; NSCLC-Anagnostou, *n* = 74, *P* = 0.02 for pTMB and *P* = 2.0 × 10^−3^ for TMB; Mann–Whitney *U*-test; Extended Data Fig. 8).

As tumor aneuploidy has been associated with inferior outcomes to ICB^14^, we investigated whether pTMB has an incremental value over tumor aneuploidy and WGD events in predicting therapeutic response. Tumor aneuploidy (Mann–Whitney *U*-test *P* = 0.35, *P* = 0.73, *P* = 0.35, *P* = 0.07, *P* = 0.50 for the NSCLC-Anagnostou, NSCLC-Shim, melanoma, mesothelioma and HNSCC cohorts, respectively) or WGD alone (Fisher’s exact *P* = 0.43, *P* = 0.73, *P* = 0.11, *P* = 0.23, *P* = 0.48 for the NSCLC-Anagnostou, NSCLC-Shim, melanoma, mesothelioma and HNSCC cohorts, respectively) failed to predict ICB response in all cohorts (Fig. 3g).

**Fig. 4:**
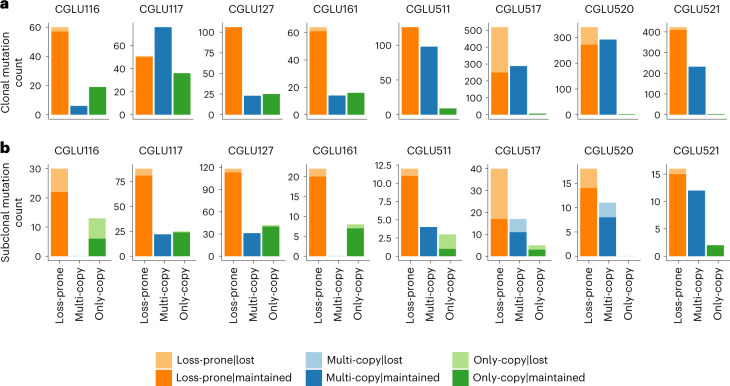
Persistent mutations are retained during cancer evolution under selective pressure of ICB. **a**, The presence of loss-prone, multi-copy and only-copy mutations identified in pre-ICB treatment NSCLC tumors was evaluated post-ICB therapy in tumors from eight patients with NSCLC and durable clinical benefit from ICB. Serial tumor samples were acquired with a minimum time difference of 6 months between biopsies and were analyzed by means of whole exome sequencing. A marked difference in the frequency of loss between clonal loss-prone and persistent mutation sets was observed, with an odds ratio of 61.46 (*P* < 2.2 × 10^−16^). Across 16 serially biopsied tumors from 8 patients, a total of 363 out of 2,836 clonal mutations that were detected in the baseline tumors were lost in the descendent tumors; of these 358 (98.6%) were clonal loss-prone mutations. In six patients analyzed, we did not identify any clonal persistent mutation that was lost in the descendent tumor. In two patients, we detected four clonal multi-copy mutations that were not detected in the descendent tumors, resulting in an extremely low rate of loss in this mutation category (clonal multi-copy mutations: 4 out of 1,031 lost, 0.4% loss frequency; clonal only-copy mutations: 1 out of 117 lost, 0.9% loss frequency). **b**, While the loss frequency was higher for subclonal loss-prone compared to persistent mutations, this did not reach statistical significance (odds ratio = 1.24, *P* = 0.44). Notably, the loss frequency for subclonal multi-copy mutations was 9.3% compared with 14.8% for subclonal loss-prone mutations, potentially indicating differential selection pressures for these alterations.

To establish the biological plausibility of persistent mutations in the context of tumor evolution, we performed whole exome sequencing analyses of serial tumor samples before and after ICB. We hypothesized that clonal persistent mutations would not be eliminated under the selective pressure of immunotherapy, as they are unlikely to undergo subclonal elimination in the context of therapy and also are unlikely to be lost by chromosomal deletions (potentially lethal in the case of mutations residing in single-copy regions and biologically implausible in the multiple-copy regions). Consistent with our hypothesis, in analyzing pre-treatment and post-acquired resistance NSCLCs from eight ICB-treated patients (Methods and Supplementary Tables 8, 12 and 13), we discovered a marked difference in the frequency of loss between clonal persistent and loss-prone mutations. A total of 363 out of 2,836 clonal mutations that were detected in the baseline tumors were lost in the descendent tumors. Of these, the vast majority were clonal loss-prone mutations (358 out of 363, 98.6%). In six out of eight patients analyzed, no clonal persistent mutation was lost in the descendent tumor, and of the two remaining patients, each had two clonal multi-copy mutations that were not detected in the descendent tumor, suggesting an extremely low rate of loss (clonal multi-copy mutations: 4 out of 1,031 lost (0.4%), clonal only-copy mutations: 1 out of 117 lost (0.9%); Fig. 4), and an odds ratio for the loss frequency of clonal loss-prone versus persistent mutations of 61.43 (*P* < 2.2 × 10^−16^; Supplementary Table 14). These analyses supported the robustness and biological basis of persistent mutations that are retained in the course of tumor evolution.

**Fig. 5:**
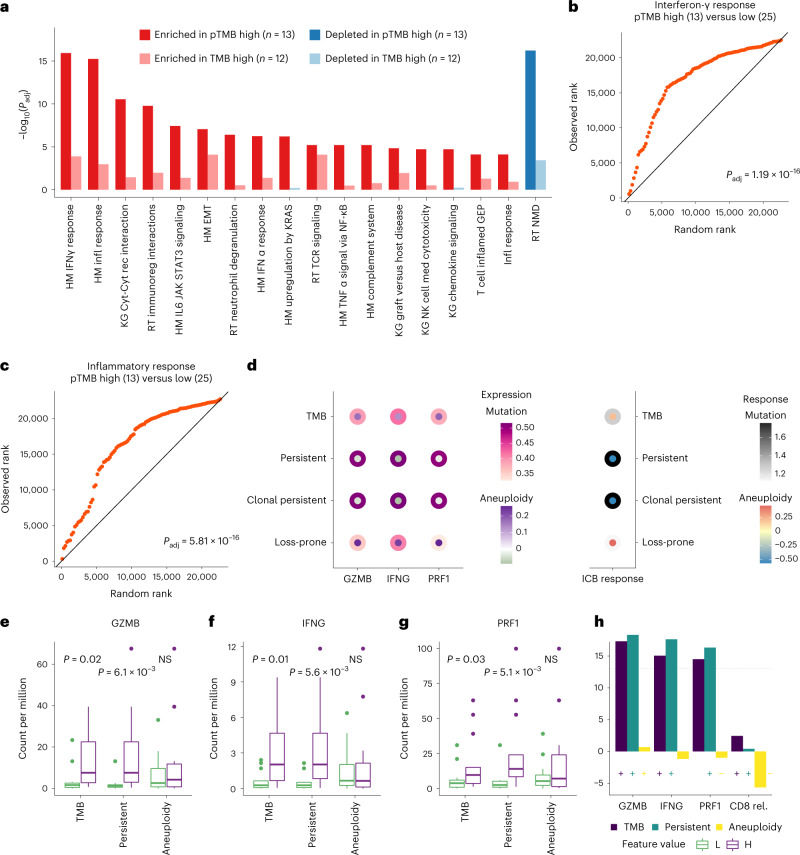
pTMB is associated with an inflamed TME in ICB-treated melanomas. **a**, Gene set enrichment analysis revealed a marked enrichment in interferon-γ (IFN-γ) response and adaptive immunity gene sets in ICB-treated melanomas with high versus low pTMB, assessed before immunotherapy initiation. In contrast, a considerably lower enrichment in inflammatory gene sets was observed in the TME of tumors stratified by their overall TMB. Nominal two-sided *P* values are calculated using permutation testing, and FDR-adjusted *P* values shown for gene set differential expression are provided for comparison of pTMB/TMB-high versus -low groups. **b**,**c**, A prominent upregulation of IFN-γ (**b**) and inflammatory response (**c**)-related gene expression programs was noted in the TME of pTMB-high melanomas. Quantile–quantile plots were generated to visually compare the ranks of genes in the pathway with ranks that were sampled from a discrete uniform distribution. **d**, pTMB counteracts the negative impact of aneuploidy on cytolytic activity and response to ICB (GZMB, IFNG, PRF1: *β*_pTMB_ = 0.5, *P* < 3 × 10^−3^; *β*_aneuploidy_ in [−0.09, −0.03], *P* > 0.05; ICB response, *β*_pTMB_ = 1.8, *P* = 4.0 × 10^−3^; *β*_aneuploidy_ = −0.56, *P* = 0.21; *β*: logistic regression model coefficient, two-sided *P* values are calculated assuming normally distributed test statistic). **e**, A greater difference in cytolytic activity was observed between tumors of high (*n* = 13) versus low (*n* = 13) pTMB, compared with TMB and aneuploidy, as evaluated by expression of GZMB (TMB *P* = 0.02, pTMB *P* = 6.1 × 10^−3^, aneuploidy *P* = 0.80). **f**, **g**, Similarly, IFNG (**f**) and PRF1 (**g**) expression was significantly higher in pTMB-high tumors compared to TMB and was not different in tumors based on their aneuploidy content (IFNG: TMB *P* = 0.01, pTMB *P* = 5.6 × 10^−3^, aneuploidy *P* = 0.88; PRF1: TMB *P* = 0.03, pTMB *P* = 5.1 × 10^−3^, aneuploidy *P* = 0.69; MW *U*-test; two-sided). **h**, No significant difference in relative abundance of CD8 T cells was observed for TMB, pTMB or aneuploidy. Box plots indicate the median value and hinges correspond to the first and third quartiles. The whiskers extend from the corresponding hinge to the furthest value within 1.5 × interquartile range from the hinge. HM, hallmark; KG, KEGG; RT, reactome; Cyt, Cytokine; Rec, receptor; EMT, epithelial–mesenchymal transition; Med, mediated; FC, fold-change; NS, non-significant; L, low; H, high; rel., relative. Source data

**Extended Data Fig. 9:**
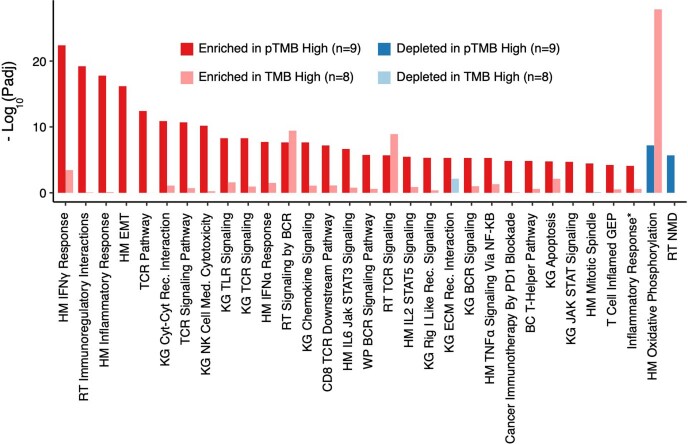
Transcriptomic profiling of on-immunotherapy melanomas reveals an upregulation of inflammatory pathways in tumors harboring a high pTMB. Gene set enrichment analyses of on-treatment pTMB-high (n = 9) vs. pTMB-low (n = 22) melanomas revealed a marked enrichment of interferon-γ and inflammatory responses in the tumor microenvironment of pTMB-high vs pTMB-low tumors after ICB. In contrast, this profile was not observed in the tumor microenvironment of TMB-high (n = 8) compared to TMB-low (n = 23) melanomas. Pathways with a minimum adjusted p-value of 1e-05 in pTMB high vs low comparison are shown. The T Cell Inflamed GEP gene set was derived from Cristescu et al., *Science*, 2018 and the Inflammatory Response gene set was derived by Ayers et al., *J Clin Invest*, 2017, while the remainder of gene sets were included in the Molecular Signatures Database (Methods). Nominal two-sided p-values are calculated using permutation testing and FDR adjusted p-values shown for gene set differential expression are provided for comparison of pTMB/TMB-high vs low groups. Abbreviations; BC: Biocarta, HM: Hallmark, KG: KEGG, RT: Reactome, EMT: Epithelial–mesenchymal transition, Cyt: Cytokine, Rec: Receptor, Med: Mediated.

Shifting our focus from the tumor to the TME, we explored transcriptomic profiles in the TME of ICB-treated tumors and postulated that a high pTMB would generate an un-interrupted feed of neoantigens which would in turn trigger interferon-γ signaling and adaptive immunity cascades that may be further enhanced with ICB. Serial RNA sequencing (RNA-seq) analyses of ICB-treated melanomas^9^ revealed a marked enrichment in interferon-γ and inflammatory response-related gene sets before therapy (Fig. 5a–c) which was significantly enhanced during ICB for high-pTMB tumors (Extended Data Fig. 9 and Supplementary Table 15). Notably, the differential enrichment in pro-inflammatory gene sets was lessened in tumors stratified by their TMB content (Fig. 5a, Extended Data Fig. 9 and Supplementary Table 15). Similar trends were observed in transcriptomic analyses comparing the TME of melanomas in the TCGA set that were stratified by pTMB (Supplementary Fig. 3 and Supplementary Table 16). We also found positive correlations between pTMB and CD8 and CD4 T cell abundances (Supplementary Fig. 4 and Supplementary Table 17) and the ratio of M1 to M2 macrophages in both baseline (Spearman’s *ρ* = 0.36) and on-ICB tumors (Spearman’s *ρ* = 0.28). Next, we investigated the relationship of pTMB, tumor aneuploidy and cytolytic activity (Methods). We found a higher expression of cytolytic markers in pTMB-high compared with TMB-high or aneuploidy-low tumors in both the TCGA (TMB, *P* > 0.05 for all genes; pTMB, *P* = 0.02 for GZMK, IFNG and PRF1; *P* = 0.04 for NKG7; aneuploidy, *P* > 0.05 for all genes; Supplementary Fig. 5) and ICB melanoma cohorts (GZMB: TMB *P* = 0.02, pTMB *P* = 6.1 × 10^−3^, aneuploidy *P* = 0.80; IFNG: TMB *P* = 0.01, pTMB *P* = 5.6 × 10^−3^, aneuploidy *P* = 0.88; PRF1: TMB *P* = 0.03, pTMB *P* = 5.1 × 10^−3^, aneuploidy *P* = 0.69; Fig. 5). In modeling the expression of key cytolytic genes based on pTMB and aneuploidy, we found that a high pTMB counteracts the negative (but not statistically significant) impact of aneuploidy on cytolytic activity and ICB response (Fig. 5 and Supplementary Fig. 6).

**Extended Data Fig. 10:**
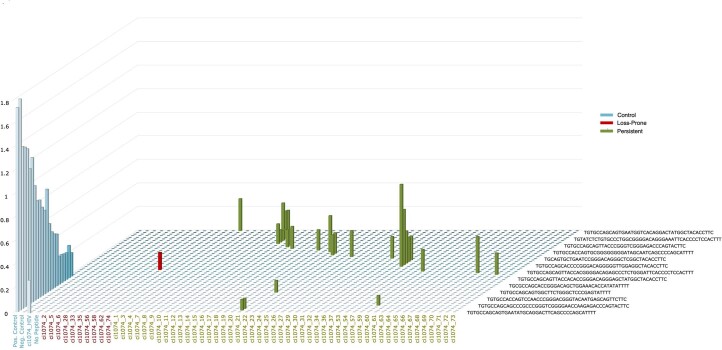
Neoantigen-specific T-cell expansion of persistent mutations. The MANAFEST assay was used to test pMANA-specific T-cell recognition. A collection of 58 neopeptides arising from 41 somatic persistent and loss-prone mutations, selected blindly with respect to pMANA status, were synthesized and tested in autologous T cell cultures from peripheral blood for patient CGLU310. A higher frequency of antigen-specific T-cell expansion was identified in neopeptides associated with persistent (green bars, 15 out of 48) vs loss-prone (red bar, 1 out of 10) mutations. T-cell clonotypes (as nucleotide sequence) and neopeptides are listed alongside the plane while the height of the bars indicates the abundance of TCR clones. A floor value of frequency of 0.08 informed by the values observed in the negative control condition was applied to TCR clonotypic abundances.

Taken together, our analyses suggested that a high pTMB, which comprises a biologically relevant measure of tumor foreignness within the overall TMB, may represent an ‘uneditable’ target set for adaptive immune responses (Extended Data Fig. 1). This hypothesis relies on the basis that pMANAs are less likely to be eliminated by chromosomal loss due to the intrinsic fitness cost to the tumor and therefore may mediate sustained neoantigen-driven immune responses and long-term clinical benefit. Similar to pTMB, pMANA load and, importantly, expressed pMANA load distinguished responding from nonresponding tumors (melanoma, *n* = 42, pTMB *P* = 2.51 × 10^−4^, pMANA *P* = 3.37 × 10^−4^, expressed pMANA *P* = 2.91 × 10^−4^; NSCLC, *n* = 74, pTMB *P* = 1.26 × 10^−4^, pMANA *P* = 1.10 × 10^−4^, expressed pMANA *P* = 1.15 × 10^−4^; Mann–Whitney *U*-test; Supplementary Table 18). We did not observe a further improvement of pMANA performance by restricting our analyses to the subset of MANAs with computationally inferred high MHC class I binding affinity^15^, which is highlighting the limitations with MANA-predicting algorithms in identifying biologically relevant neoepitopes. To this end, we sought to generate additional functional proof that pMANAs are indeed recognized and elicit epitope-specific T cell expansions and pulsed autologous T cells from a patient with NSCLC with peptides derived from persistent and loss-prone mutations. All but one of the peptides that elicited T cell receptor (TCR) clonotypic expansions were encoded by persistent mutations (Extended Data Fig. 10 and Supplementary Tables 19 and 20), suggesting that pMANAs are recognized by CD8^+^ T cells and further supporting the biological importance of persistent mutations.

## Discussion

Since the first reports recognizing TMB as a predictor of ICB response for patients with melanoma and NSCLC^16,17^, it has become clear that TMB as a numeric value or binarized feature can only partially predict therapeutic response. Our findings suggest that a high pTMB, a biologically relevant measure of tumor foreignness within the overall TMB, represents an ‘uneditable’ target set for adaptive immune responses and may function as an intrinsic driver of sustained immunologic tumor control that cannot be readily bypassed by neoantigen loss via chromosomal deletions during cancer evolution.

Similar to TMB, which is linked with response to immunotherapy in a dose-dependent and cancer lineage-specific manner^18^, pTMB has to be considered in the context of the background aneuploidy rate within a specific tumor type. What we have learned from the increasing number of studies evaluating the overall TMB in predicting ICB response is that using a fixed pan-cancer threshold for a biomarker with different distributions and dynamic ranges depending on cancer lineage^19^ can be challenging and may misevaluate up to 25% of ICB-responsive tumors^20^. In our current work and to avoid these challenges, we evaluated both persistent mutations and TMB as continuous variables in the context of ICB response; thus, our findings are less susceptible to artefactual associations resulting from application of a threshold. To expand our analyses in supporting a biologically distinct role for pTMB which is reflected in therapeutic response difference compared with overall TMB-based classifications, we evaluated the number of tumors with differential pTMB/TMB classification within the melanoma ICB cohort and found a higher response rate among tumors reclassified by their pTMB content. Notably, the relative contribution of multi- and only-copy mutation components to the overall pTMB varies across cancer lineages. This pattern appears to be driven by the dominant copy number state of the tumor, suggesting that the dominant copy number state has to be considered together with the sequence alteration load affecting these genomic regions.

The premise of pTMB relies on the potential of pMANAs to mediate sustained neoantigen-driven immune responses. Overall MANA burden has failed to demonstrate an incremental value over TMB in predicting clinical outcomes with ICB, as it is the neoantigen quality and not the quantity which may be most informative in predicting therapeutic response^21–23^. Another feature that may determine the role of MANAs in anti-tumor immune responses is expression, as expressed single-base substitution-derived neopeptides have been shown to more accurately predict response to ICB compared with TMB^9^. In line with this notion, the significance of mutant protein abundance in driving T cell responses may further support the importance of multi-copy persistent mutations, as their presence at higher numbers of copies per cell likely correlates with higher expression of mutant messenger RNAs and proteins. We indeed found a marginal improvement in outcome prognostication of pMANA burden compared with pTMB in ICB-treated NSCLC. Importantly, by testing pMANA-specific TCR clonotypic expansions in vitro, we provide proof that pMANAs can elicit memory T cell responses that are likely to drive tumor elimination.

Placing pTMB in the context of other genomic features that have been associated with response to ICB^1^, we assessed the clonal architecture of persistent mutations and considered the potential confounding effect of tumor clonal heterogeneity. Overall, in the TCGA and ICB cohorts, the cellular fractions of persistent mutations did not differ from loss-prone mutations; notably, persistent mutations in the multi-copy category tended to be more clonal in a cancer lineage-dependent manner, suggesting that these may have been acquired earlier in tumor evolution before the copy number gain event. Persistent mutations more optimally distinguished responding from nonresponding tumors compared with clonal TMB in all ICB cohorts analyzed. Importantly, clonal persistent mutations were more significantly associated with response in the ICB-treated NSCLC cohorts. These findings suggest that considering pTMB and clonal heterogeneity together may be most informative in predicting response to immunotherapy.

While pTMB is related to tumor aneuploidy and a higher degree of large-scale chromosomal changes has been reported in ICB nonresponsive tumors^14^, in our analyses tumor aneuploidy alone failed to differentiate responding from nonresponding tumors. While our analyses did not show a strong association between tumor aneuploidy or WGD and response to ICB as individual predictive biomarkers, the number of persistent mutations was correlated with tumor aneuploidy, and we found an enrichment for multi-copy persistent mutations in tumors with WGD. Our findings highlight the importance of measuring mutational burden in regions of the genome with structural changes rather than considering overall TMB or tumor aneuploidy independently.

Importantly, we studied the evolution of persistent mutations in the evolutionary trajectories shaped by selective pressure of ICB. We hypothesized that multi-copy mutations would inherently be more difficult to lose, as the process of loss would require multiple distinct genomic events and, similarly, single-copy mutations are unlikely to be lost by chromosomal deletions as these may be detrimental to the cancer cell, rendering persistent mutation loss biologically improbable. Consistent with this notion, we discovered that persistent mutations were retained in the context of tumor evolution while losses predominantly affected loss-prone mutations. While approximately 99% of mutations lost in serial analyses of NSCLC during ICB were loss-prone mutations, four clonal multi-copy persistent mutations were not detected in comparative analyses of baseline/ICB-resistant tumors, which may be explained by the presence of multiple copies of a mutation in tandem or in close proximity on a common chromosomal segment; in these cases, loss of multiple copies could be achieved by a single genomic event.

Taken together, our findings suggest that mutations located in single-copy regions or those present in multiple copies in the cancer genome are unlikely to be lost under the selective pressure of immunotherapy due to the intrinsic fitness cost to the tumor, and therefore may serve as a key driver of sustained immunologic tumor control.

## Methods

### Cohorts

We evaluated 10,742 tumor samples from TCGA and 485 NSCLC, melanoma and mesothelioma tumor samples from published cohorts of patients that received ICB^2,4,6–8,10,24^ (Supplementary Table 8). Tumors across 31 tumor types were analyzed from TCGA (ACC: Adrenocortical carcinoma, BLCA: Bladder Urothelial Carcinoma, BRCA: Breast invasive carcinoma, CESC: Cervical squamous cell carcinoma and endocervical adenocarcinoma, CHOL: Cholangiocarcinoma, COAD: Colon adenocarcinoma, ESCA: Esophageal carcinoma, GBM: Glioblastoma multiforme, HNSC: Head and Neck squamous cell carcinoma, KICH: Kidney Chromophobe, KIRC: Kidney renal clear cell carcinoma, KIRP: Kidney renal papillary cell carcinoma, LGG: Brain Lower Grade Glioma, LIHC: Liver hepatocellular carcinoma, LUAD: Lung adenocarcinoma, LUSC: Lung squamous cell carcinoma, MESO: Mesothelioma, OV: Ovarian serous cystadenocarcinoma, PAAD: Pancreatic adenocarcinoma, PCPG: Pheochromocytoma and Paraganglioma, PRAD: Prostate adenocarcinoma, READ: Rectum adenocarcinoma, SARC: Sarcoma, SKCM: Skin Cutaneous Melanoma, STAD: Stomach adenocarcinoma, TGCT: Testicular Germ Cell Tumors, THCA: Thyroid carcinoma, THYM: Thymoma, UCEC: Uterine Corpus Endometrial Carcinoma, UCS: Uterine Carcinosarcoma, UVM: Uveal Melanoma). Patients with melanoma across four source studies^6–8,22^ were combined to generate an aggregated melanoma cohort (*n* = 202). Clinical outcomes were retrieved from the original publications (Supplementary Table 8). We further performed whole exome sequencing analyses for a cohort of 39 patients with HPV-negative HNSCC who received ICB at the University of Chicago (HNSCC cohort). We assessed serially sampled NSCLC tumors from four patients from a published study^3^ as well as from four patients with NSCLC who received ICB at the Nederlands Kanker Instituut (NKI set). The studies were conducted in accordance with the Declaration of Helsinki and were approved by the Nederlands Kanker Instituut Institutional Review Board (IRB) and the University of Chicago Comprehensive Cancer Center IRB (8980). All patients provided written, informed consent for sample acquisition for research purposes; their participation in the protocol was not compensated. Clinical and pathologic characteristics for all patients, as well as self-reported sex and self-reported race, are summarized in Supplementary Table 4.

### Whole exome sequencing and sequence data processing

DNA extraction and genomic library preparation were performed following manufacturers’ protocols^2^. The coding sequences were captured in solution using the SureSelect XT Human All Exon V6 kit in the HNSCC cohort, and using the SureSelect Human All Exon V4 kit in the NKI cohort. Whole exome sequencing-derived multi-center mutation calls from the TCGA pan-cancer atlas^25^ were retrieved from the NCI Genomic Data Commons (https://gdc.cancer.gov/about-data/publications/mc3-2017) and filtered to keep nonsynonymous alterations. For the ovarian cancer (OV) tumor type, indels were excluded from all downstream analyses to minimize technical artifacts^26^. Somatic copy number profiles including estimates of tumor purity and ploidy, as well as allele-specific copy number states^27^, were acquired via the pan-cancer atlas (https://gdc.cancer.gov/about-data/publications/pancanatlas). Clinical annotations of tumors were accessed using the TCGA clinical data resource^28^. For the HNSCC subset in TCGA, HPV status was retrieved from cBioPortal (https://www.cbioportal.org/study/summary?id=hnsc_tcga_pan_can_atlas_2018). Analyses of copy number profiles to establish the background rate of genomic loss were performed on 10,742 tumor samples where the segmental allele-specific copy numbers were available. For a subset of 9,242 tumor samples from the above, both somatic mutation calls and copy number profiles were available, enabling assessment of persistent mutations. For a subset of 8,925 tumors where clinical data annotations including overall survival and tumor stage assessment were available, survival analyses evaluating the contribution of persistent mutations were performed.

For the publicly available published ICB cohorts, we retrieved allele-specific copy number profile, tumor purity and ploidy estimates, as well as somatic mutation calls, raw gene expression counts and clinical annotations of response to treatment from the original publications. Furthermore, for the Riaz et al. melanoma cohort^6^, allele-specific somatic copy number profile and tumor purity and ploidy estimates were generated by application of FACETS (v.0.6.0) to tumor and matched normal sequence data^29^. For the Liu et al. melanoma cohort^7^, the short-read archive files were accessioned from the Sequence Read Archive (SRA) and this sample set was filtered to keep only tumors with no previous anti-CTLA4 treatment. Adapter sequences were detected and trimmed using FASTP (v.0.20.0)^30^. Sequenced reads were aligned to the reference genome assembly hg19 using bowtie2 (v.2.3.5)^31^, and duplicate reads marked by sambamba (v.0.8)^32^. Tumor purity and ploidy estimates, as well as somatic copy number profiles, were derived by application of FACETS^29^ to tumor and matched normal pairs. For the Hugo et al. melanoma cohort^8^, fastq files were obtained from the SRA. Sequencing read processing and alignment were performed as described for the Liu et al. cohort, and copy number profiles were similarly obtained by application of FACETS. For the Shim et al. NSCLC cohort^10^, somatic mutations were narrowed down to those with mutant allele fraction greater than or equal to 10% to minimize sequencing artifacts. Tumor purity and ploidy estimates, and somatic copy number profiles, were generated by application of FACETS^29^ to tumor and matched normal pairs. For the HNSCC cohort, somatic mutations were identified using the Strelka mutation calling pipeline (v.2.9.10)^33^. Mutations in common single nucleotide polymorphism (SNP) locations (dbSNP v.138) and with greater than one BLAT^34^ hit were filtered out. The final set of mutations were obtained after filtering for tumor mutant allele fraction ≥ 10%, normal mutant allele fraction ≤ 3% and matched normal coverage ≥ 11×. For samples from the NKI set, sequence read processing and alignment were performed similarly to the samples from the Liu cohort. Tumor purity and ploidy estimates and somatic copy number profiles were derived by application of FACETS. While we did not have uniform documentation of microsatellite instability (MSI) status in the ICB cohorts analyzed, the very low background prevalence of MSI-high tumors in NSCLC (<1%), melanoma (<1%), mesothelioma (~2%) and HNSCC (<1%)^35^ renders MSI an unlikely confounder in this study.

### Evaluation of mutation multiplicity and cancer cell fraction

Mutation cellular fractions were estimated as follows^3,36^: considering the tumor sample purity *α*, tumor copy number *n*_T_ and normal copy number *n*_N_, the expected variant allele fraction *V*_exp_ for a mutation at cellular fraction *C* with multiplicity *m* (that is, *m* mutant copies per cancer cell) can be calculated as:

$$V_{\mathrm{exp}} = \frac{{m\;C\;\alpha }}{{\alpha \;n_{\mathrm{T}} + \left( {1 - \alpha } \right)n_{\mathrm{N}}}}$$

The purity and segmental tumor and normal copy numbers were determined via genome-wide analysis of sequencing coverage distribution and b-allele frequency of heterozygous SNPs in each cohort. Assuming a binomial distribution for the number of reads harboring the mutant allele, a 95% confidence interval (CI) is constructed for *V*_exp_ using the distinct total coverage and mutant read counts for each mutation (that is, coverage and read counts after exclusion of reads marked as duplicates). Since estimates for *α*, *n*_T_ and *n*_N_ are available, this yields a 95% CI for the product of mutation at cellular fraction *C* and multiplicity *m*. By application of the following rules, one can derive estimates for *C* and *m*: (1) If the CI for *mC* contains an integer, the mutation is deemed clonal and that value is assigned to the multiplicity. (2) If the entire CI is below 1, multiplicity is assumed to be 1 and the mutation is subclonal, except cases where it is within a tolerance threshold of 1 (*C* > 0.75). (3) For a CI that is entirely above 1 and does not include any integer, *m* is greater than 1 and is assigned such that the CI falls within the expected range [0,1]. Mutation clonality can now be calculated using the rule in (2).

### Assessment of single-copy, multi-copy and persistent TMB

The nonsynonymous somatic mutations in each tumor were intersected with the segmental integer copy number profile to assign minor and major copy number states to the mutated loci. Mutation multiplicity (number of mutated copies per cell) and cancer cell fraction (proportion of cancer cells harboring the mutation) were estimated based on the mutant read count, total coverage, tumor purity, and the major and minor allele-specific copy numbers in the tumor and normal compartments for each mutation. Mutations present in more than one copy per cancer cell constituted the multi-copy category. Those present in regions of the genome with a single copy (total copy number = 1) were included in the single-copy category. The pTMB was defined as the number of mutations in either the multi-copy or single-copy category. For mesotheliomas, given the predominance of copy number losses^4,37^, the persistent mutation burden was limited to mutations within single-copy regions of the genome. Furthermore, to assess the differential potential of persistent mutation in predicting outcome compared with TMB, we defined the number of loss-prone mutations in each tumor sample as the difference between the total number of mutations assessed (excluding mutations on sex chromosomes or those at loci without copy number assignment) and the number of persistent mutations. Finally, to achieve harmonized comparisons, mutations on sex chromosomes or on loci lacking allele-specific copy number assignment were excluded from analyses.

### Characterization of tumor aneuploidy

Aneuploidy metrics were calculated for tumor samples from TCGA and ICB cohorts, and their relationship with persistent mutation burden was characterized. Furthermore, in ICB-treated cohorts, aneuploidy metrics were also considered as independent predictors of outcome. The fraction of genome with allelic imbalance was calculated as a broad metric summarizing the aneuploidy level across the autosomes. We also considered the fraction of genome with single copies (total copy number of 1) and the fraction of genome with multiple copies (that is, major allele-specific copy number greater than 1), given their direct link to persistent mutation burden. Tumor samples with more that 50% of the autosomal length at major allele-specific copy number of 2 or above were marked as having undergone WGD^38^.

### Evaluation of the background rate of genomic loss

To evaluate the background rate of genomic loss, we analyzed the somatic copy number profiles of 10,742 samples from TCGA. In each tumor sample, the chromosome arms in diploid state were defined as those where 75% of the length of segments covering the arm was copy neutral (total copy number of 2) and did not harbor LOH. The chromosome arms in haploid state had 75% of their length covered by segments with a total copy number of 1. The rate of loss in diploid regions of the genome was defined as *R*_D_:

$$R_{\mathrm{D}} = \frac{{2 \times \;l_{\mathrm{D}}^{\mathrm{HD}} + l_{\mathrm{D}}^{\mathrm{HM}}}}{{2 \times \;l_{\mathrm{D}}}}$$

Where *l*_D_ indicates the total length of segments in arms of diploid state, $l_{\mathrm{D}}^{\mathrm{HD}}$ is the total length of the segments with homozygous deletion in diploid arms and $l_{\mathrm{D}}^{\mathrm{HM}}$ is the total length of the segments with single-copy loss in diploid arms. Similarly, the rate of loss in haploid regions of the genome was defined as *R*_H_:

$R_{\mathrm{H}} = \frac{{l_{\mathrm{H}}^{\mathrm{HD}}}}{{l_{\mathrm{H}}}}$Where *l*_H_ is the total length of segments in haploid arms and $l_{\mathrm{H}}^{\mathrm{HD}}$ is the total length of segments with homozygous deletion in those arms. Comparison of the background rates of genomic loss was performed on subsets of the TCGA tumor samples where at least one chromosome arm was found in each of diploid and haploid states (*n* = 5,244).

### Evaluation of pTMB quantification by gene panel-targeted NGS

To determine the feasibility of using clinical targeted NGS to estimate pTMB, we performed in silico simulations as follows. Given the inherent limitation of targeted NGS in identification of mutations in tumor types with low TMB, we performed a focused analysis in melanoma and NSCLC cohorts. We assumed that allele-specific copy number estimates could be derived by targeted NGS as previously shown^29,39^. Therefore, we focused our analysis on the subset of mutations that would be captured by the genomic intervals contained in FoundationOne CDx, which is a widely used clinical targeted NGS panel. The list of 309 genes with their full coding sequence included in the FoundationOne CDx panel was retrieved from the Food and Drug Administration website at https://www.accessdata.fda.gov/cdrh_docs/pdf17/P170019S006C.pdf. The RefSeq Select transcript set was used to determine the genomic coordinates of the coding exons for each gene. Mutations in each tumor sample were intersected with panel coordinates to determine simulated estimates of TMB and pTMB as captured by the panel.

### Differential expression and gene set enrichment analysis

Expression counts from RNA-seq of pre-treatment melanoma tumors from the CM038 melanoma cohort were retrieved from the original publication^24^. Differential expression testing was performed using DESeq2 (ref. 40) and the resulting *P* values were corrected for multiple testing using the Benjamini–Hochberg procedure. For the TCGA tumor type SKCM, the TCGAbiolinks (v.2.25.0) R package^41^ was used to download harmonized raw RNA-seq counts data from the NCI Genomic Data Commons within the target cancer type. This sample set was then narrowed down to the set of samples with persistent mutation estimates and available overall survival data, and comparisons were performed between samples within the top tertile of pTMB/TMB-informed risk (high risk) versus the remaining set (low risk). For gene set enrichment analysis, each gene that passed the count threshold was ranked by ‘-log(p) * sign(fc)’, where p is *P* value and fc is fold-change, resulting in ranking where the genes on each flank represent the mostly statistically significantly up- or down-regulated genes and the genes in the middle are the least significant. Gene set enrichment analysis (gsea) was then performed using the fgsea (v.1.20.0) R package^42^ with a curated list of gene sets from the Molecular Signatures Database related to immune responses and cancer hallmarks. Tumors were classified into high or low groups for TMB and pTMB using the second tertile value. The complete list of gene sets can be found in Supplementary Table 15, which contains the gsea results for comparisons based on pTMB and TMB in baseline and on-treatment samples. The *P* values for gsea were corrected for multiple testing with the Benjamini–Hochberg procedure. Quantile–quantile plots were made to provide a visual comparison of the ranks of pathway genes with a set of ranks sampled from the background distribution.

### Modeling of cytolytic activity

Gene level expression values (in counts per million) were used from the CM038 IO melanoma cohort^24^ and the TCGA melanoma cohort. In each cohort, expression levels for a selected set of gene markers of cytolytic activity were compared between tumors in the top and bottom tertiles of a number of key variables of interest using Mann–Whitney *U*-test. Furthermore, a multivariable linear regression model defined the combined contribution of a mutation-based marker (that is, pTMB, TMB and so on) and aneuploidy (as measured by the fraction of genome with allelic imbalance) to cytolytic activity. Briefly, both mutation-based marker values and gene expression levels were pseudo log-transformed to control the right skew in the distribution. Next, each variable was scaled to have zero mean and unit variance over the analyzed cohort. In each regression model, predictor coefficients and the associated *P* values were recorded. In the IO melanoma cohort, multivariable logistic regression was used to model the contribution of mutation-based markers and aneuploidy. In addition, estimates for the relative abundances of 22 immune cell subpopulations derived by CIBERSORT v.1.06 were retrieved from the earlier publication. For the TCGA melanoma tumors, the relative abundances of CD8 T cells were retrieved from the genomic data commons^43^.

### Longitudinal tracking of persistent mutations

For the eight patients with NSCLC with serially biopsied tumor samples, tumor samples were acquired before ICB and at the time of acquired resistance; for all cases, a minimum of 6 months lapsed between ICB initiation and re-biopsy in the setting of acquired resistance. For each patient, the set of mutations identified in the baseline sample was annotated with distinct total coverage, distinct mutant read count, and minor and major allele-specific copy numbers. These annotations were combined with the estimated purity of the tumor sample to yield estimates of mutation cancer cell fraction and multiplicity. The combination of copy number assignment and multiplicity estimate for each mutation in the baseline sample enabled identification of only-copy, multi-copy and persistent mutations, as well as those prone to loss (loss-prone). Mutations identified in the baseline sample with mutant allele fraction of zero at the time of progression were deemed lost (Supplementary Tables 12 and 13).

### Analysis of mutation-associated neoantigens

An integrated analysis of persistent mutation-associated neoantigens was performed in the ICB NSCLC^2^ and melanoma^24^ cohorts. Briefly, MANA predictions by ImmunoSelect-R pipeline (Personal Genome Diagnostics) were retrieved from the original studies. The set of predicted peptides was restricted to those with predicted MHC class I binding affinity (half-maximum inhibitory concentration (IC_50_)) less than 500 nM, and mutations with at least one associated peptide were marked as MANA-encoding. Mutations in genes with nonzero median expression in the respective TCGA tumor type were marked as expressed.

### Assessment of replication timing for persistent mutations

We compared persistent and loss-prone mutations with regard to the replication timing of the mutated loci in melanoma (TCGA-SKCM) and NSCLC (TCGA-LUAD, TCGA-LUSC) tumors from TCGA. We retrieved replication timing scores for NHEK (skin) and IMR90 (lung) cell lines, which were measured by Repli-Seq methodology as part of the ENCODE project, using the UCSC Table Browser. In each cell line, we used scores from the ‘wavlet-smoothed signal’ track, which is the result of application of a wavelet smoothing transformation to the weighted average of the percentage-normalized signals in 1-kilobase intervals across the genome, where higher values indicate earlier replication timing. Genomic intervals were marked based on their quintile membership, and the frequencies of persistent and loss-prone mutations across the quintiles were visualized. Replication timings of persistent and loss-prone mutations were compared by evaluating Cohen’s *d* effect size.

### Analysis of NGS technical limitations

To assess the possibility of technical artifacts preferentially impacting the somatic mutation calls in our pan-cancer analysis of 31 tumor types, we determined the prevalence of persistent and loss-prone mutations identified in regions of the genome susceptible to limitations of NGS analysis. The UCSC Table Browser was used to retrieve the ‘Problematic Regions’ track, including regions marked by ENCODE^12^, Genome-In-A-Bottle^13^ and NCBI GeT-RM^44^.

### Functional T cell assays

The MANAFEST (Mutation-Associated NeoAntigen Functional Expansion of Specific T Cells) assay^3^ was employed to determine MANA-specific T cell clonotypic expansions in the peripheral blood of a patient with NSCLC who attained long progression-free and overall survival with ICB (CGLU310). Briefly, candidate neopeptides (JPT Peptide Technologies; Supplementary Table 19) were synthesized and each used to stimulate and co-culture T cells in vitro. On day 10, cells were collected and the CD8^+^ fraction was isolated using a CD8^+^ negative enrichment kit (EasySep, STEMCELL Technologies), followed by DNA extraction from each CD8-enriched culture condition. TCR Vβ CDR3 sequencing was performed by the Sidney Kimmel Comprehensive Cancer Center (SKCCC) FEST and TCR Immunogenomics Core (FTIC) on genomic DNA from each T cell condition using the Oncomine TCR Beta short-read assay (Illumina)^11^. Following data pre-processing, alignment and trimming, productive frequencies of TCR clonotypes were calculated. To be considered antigen-specific, a T cell clonotype must have met the following criteria: (1) significant expansion (Fisher’s exact test with Benjamini–Hochberg correction for false discovery rate (FDR), *P* < 0.05) compared with T cells cultured without peptide; (2) significant expansion compared with every other peptide-stimulated culture (FDR < 0.05); (3) an odds ratio greater than 5 compared with all other conditions; (4) at least 30 reads in the ‘positive’ well; and (5) at least 2× higher frequency than background clonotypic expansions as detected in the HIV-negative control condition.

### Evaluation of differentially classified tumors by TMB and pTMB

Reclassification rates based on pTMB versus TMB were computed as follows: in each tumor type and for each variable (TMB and pTMB), a series of quantile values ranging from 5% to 95% in 5% increments were applied to define samples with high and low values for that variable. Next, at each quantile value, we calculated the rate at which sample classification differed by the two metrics (that is, the combined prevalence of samples that were pTMB-low, TMB-high and samples that were pTMB-high, TMB-low within that tumor type for that quantile threshold) to derive the reclassification rate. In the ICB cohorts, we determined the cases with differential pTMB/TMB classification and compared the therapeutic response rates between pTMB-low/TMB-high and pTMB-high/TMB-low tumors. For each predictor variable (pTMB or pTMB), the second tertile was used to determine tumor samples with high and low values. Given the current cohort sizes, sufficient sample size for this comparison was only available in the combined melanoma cohort where 23 samples where labeled as TMB-high/pTMB-low and another 23 samples were marked as TMB-low/pTMB-high.

### Survival analysis of TCGA tumors

The relationship of pTMB and TMB with overall survival was assessed in 8,925 patients from 31 cohorts in TCGA. Tumor types with more than 50 informative samples were analyzed and overall survival was selected as an endpoint. Briefly, for each tumor type and stage combination, Cox proportional-hazards (CoxPH) models were constructed for each one of the following features as independent continuous predictors: TMB, pTMB, clonal pTMB, multi-copy mutations, clonal multi-copy mutations, only-copy mutations, clonal only-copy mutations (continuous CoxPH model). In cases where an increase in the predictor variable was associated with better outcome (longer survival), a second CoxPH model was used to assess the difference in overall survival between tumors in the top one-third and bottom two-thirds of predicted risk (categorical CoxPH model).

### Statistical analyses

For the TCGA cohort, in each tumor type, a CoxPH model was used to evaluate the contributions of TMB and pTMB to overall survival. The predicted risk values from these models were then used to stratify the tumors into low- and high-risk groups using the second tertile of the predicted risk as the threshold. For the immunotherapy cohorts, clinical response assessments were retrieved from the original publications. The Mann–Whitney *U*-test was used to evaluate the differences of continuous variables between groups, including the differences of predictive variables between responding and nonresponding tumors, and the differences of background rate of loss between haploid and diploid regions of the genome. Cohen’s *d* statistic was used to quantify the effect size of each predictor variable in the ICB cohorts. Fisher’s exact test was used to assess the association of dichotomous variables (such as WGD) with therapeutic response. The associations of the fraction of mutations in single- and multi-copy regions with TMB ranks were evaluated by the Pearson correlation coefficient, while nonparametric correlations were evaluated by the Spearman correlation coefficient. The Kaplan–Meier method was used to estimate the survival function and the survival curves were compared using the nonparametric log-rank test. All *P* values were based on two-sided testing and differences were considered significant at *P* < 0.05. The *P* values reported were not adjusted for multiple hypothesis testing unless explicitly stated. Statistical analyses were done using R v.3.6 and higher (http://www.R-project.org/).

### Reporting summary

Further information on research design is available in the Nature Portfolio Reporting Summary linked to this article.

## Online content

Any methods, additional references, Nature Portfolio reporting summaries, source data, extended data, supplementary information, acknowledgements, peer review information; details of author contributions and competing interests; and statements of data and code availability are available at 10.1038/s41591-022-02163-w.

## Supplementary information

Supplementary Information Supplementary Figs. 1–6.

Reporting Summary

Supplementary Tables Supplementary Table 1. Background rate of genomic loss in diploid vs haploid regions of the genome in the TCGA cohorts. Supplementary Table 2. Frequencies of genome and mutations in multi-copy and single-copy states in the TCGA cohorts. Supplementary Table 3. Persistent mutation characteristics in the TCGA cohorts. Supplementary Table 4. Clinical characteristics of ICB-treated HNSCC cohort. Supplementary Table 5. Summary of next-generation sequencing analyses for the HNSCC and ICB acquired resistance cohorts. Supplementary Table 6. Summary of copy number analyses in the HNSCC and ICB acquired resistance cohorts. Supplementary Table 7. Somatic sequence alterations in the ICB-treated HNSCC cohort. Supplementary Table 8. Summary of IO cohorts analyzed. Supplementary Table 9. Multi-copy, only-copy, persistent mutations, and aneuploidy metrics in IO cohorts. Supplementary Table 10. Survival analysis in the TCGA cohorts. Supplementary Table 11. Effect size analysis for pTMB and TMB in the ICB cohorts. Supplementary Table 12. Persistent mutations in the Anagnostou et al., Cancer Discovery, 2017 acquired resistance cohort. Supplementary Table 13. Persistent mutations in the NKI acquired resistance cohort. Supplementary Table 14. Longitudinal tracking of persistent and loss-prone mutations during ICB. Supplementary Table 15. Gene set enrichment analysis of baseline and on-treatment melanomas stratified by pTMB and TMB in the CM038 cohort. Supplementary Table 16. Gene set enrichment analysis of melanomas in TCGA stratified by pTMB and TMB. Supplementary Table 17. Frequencies of immune cell populations in the TME of melanomas in the CM038 cohort. Supplementary Table 18. Association of persistent MANAs with response to ICB. Supplementary Table 19. Summary of peptides analyzed by the MANAFEST assay. Supplementary Table 20. pMANA-stimulated T cell clonotypic expansions.

Supplementary Data 1 Comparison of persistent mutation metrics in tumors with and without whole-genome doubling.

## Data Availability

Source data for the TCGA tumor samples were retrieved from http://cancergenome.nih.gov. WES-derived somatic mutation calls from the TCGA PanCancer Atlas MC3 project were retrieved from the NCI Genomic Data Commons (https://gdc.cancer.gov/about-data/publications/mc3-2017). Somatic copy number profiles (https://gdc.cancer.gov/about-data/publications/pancanatlas) and clinical data (https://gdc.cancer.gov/about-data/publications/PanCan-Clinical-2018) were accessed from Genomic Data Commons. Previously published genomic data, re-analyzed here, were obtained from the material of the original publications and from dbGaP under accession code phs000452.v3.p1 (ref. 7), and from the Sequence Read Archive (SRA) under accession codes SRP095809 (ref. 6), SRP067938 (ref. 8) and SRP090294 (ref. 8). WES sequence data for the HNSCC and NKI cohorts from patients who consented to data deposition can be retrieved from the European Genome-phenome Archive (EGA accession number EGAS00001006660) in controlled access mode, subject to approval by the Data Access Committee, for use in not-for-profit organizations, with approval of local IRB/ERB, for approved projects, by approved users, and not-for-profit use. The de-identified clinical and genomic data used for the analyses in this study are available in the supplementary tables and all publicly available data elements have been referenced. Source data are provided with this paper.

## Source data

Source Data Fig. 1 Statistical source data.
Source Data Fig. 3 Statistical source data.
Source Data Fig. 5 Statistical source data.
Source Data Extended Data Fig. 2 Statistical source data.
Source Data Extended Data Fig. 4 Statistical source data.
Source Data Extended Data Fig. 8 Statistical source data.

## Code availability

The study did not involve development of new software packages.
