# Supplementary material for: Persistent mutation burden drives sustained anti-tumor immune responses
Source: Nat Med. 2023 Jan 26;29(2):440–9. doi: 10.1038/s41591-022-02163-w (PMC9941047; doi:10.1038/s41591-022-02163-w)
Supplement: Supplementary file 1 — Supplementary Figs. 1–6. [file 41591_2022_2163_MOESM1_ESM.pdf]

nature medicine

Article

https://doi.org/10.1038/s41591-022-02163-w

Persistent mutation burden drives sustained anti-tumor immune responses

In the format provided by the authors and unedited

Supplementary Information

Persistent Mutation Burden Drives Sustained Anti-Tumor Immune Responses

Noushin Niknafs[1], Archana Balan[1], Christopher Cherry[1], Karlijn Hummelink[2], Kim Monkhorst[2], Xiaoshan M. Shao[1,3], Zineb Belcaid[1], Kristen A. Marrone[1], Joseph Murray[1], Kellie N. Smith[1,3], Benjamin Levy[1], Josephine Feliciano[1], Christine L. Hann[1], Vincent Lam[1], Drew M. Pardoll[1,3], Rachel Karchin[1,4], Tanguy Y. Seiwert[1], Julie R. Brahmer[1,3], Patrick M. Forde[1,3], Victor E. Velculescu[1,3] and Valsamo Anagnostou[1,3*]

[1]The Sidney Kimmel Comprehensive Cancer Center, Johns Hopkins University School of Medicine, Baltimore, MD 21287, USA

[2]Netherlands Cancer Institute, Amsterdam, 1066 CX, Netherlands

[3]The Bloomberg-Kimmel Institute for Cancer Immunotherapy, Johns Hopkins University School of Medicine, Baltimore, MD 21287, USA

[4]Institute for Computational Medicine, Johns Hopkins University, Baltimore, Maryland 21218, USA

*** To whom correspondence should be addressed:**

Valsamo Anagnostou, MD, PhD

CRB2, Rm546, 1550 Orleans Street

Baltimore, MD 21287

Phone: 410-614-8948

Email: vanagno1@jhmi.edu

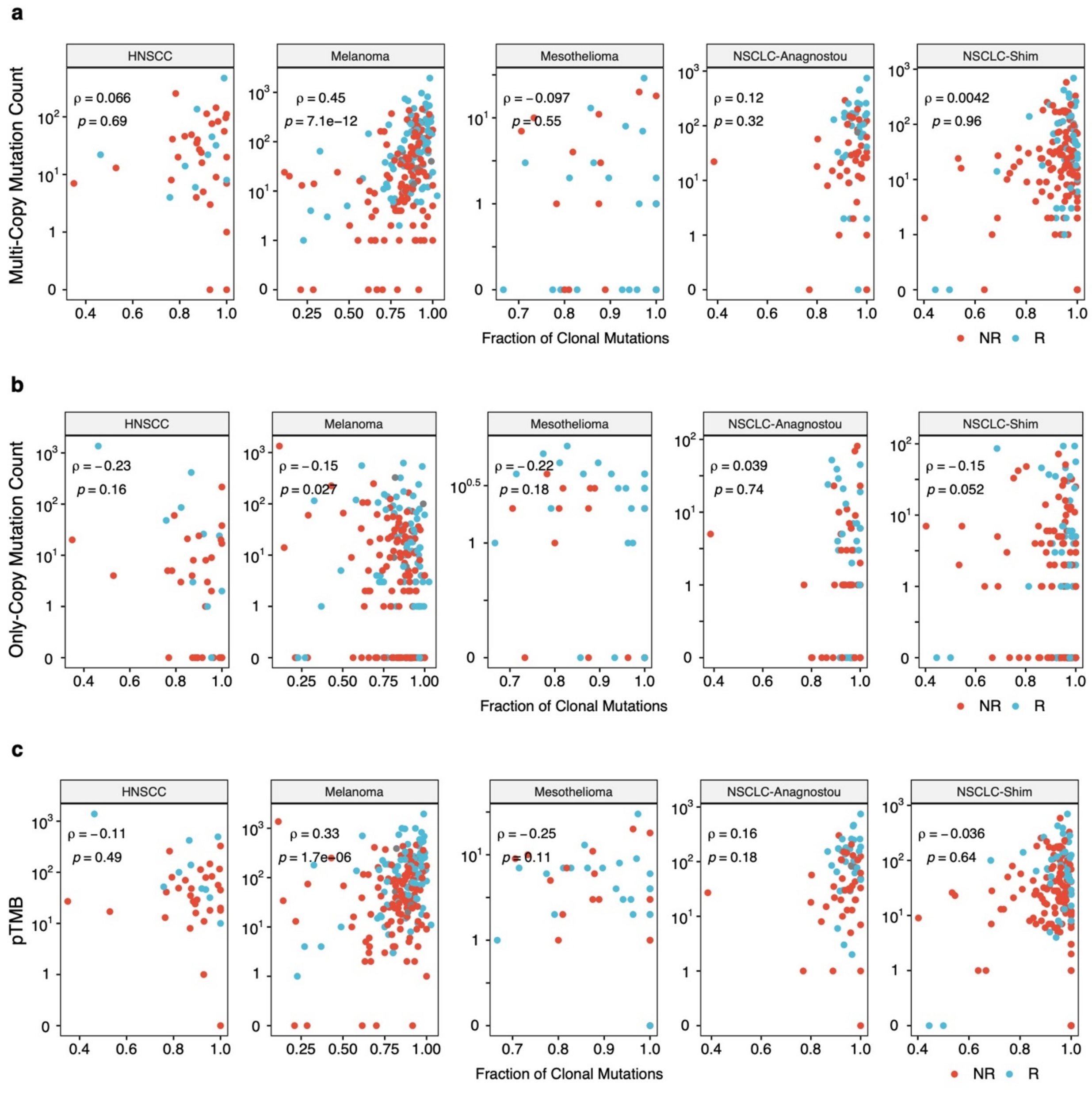

Supplementary Fig.1 Association of intra-tumor clonal heterogeneity with persistent mutations. The correlation between the fraction of clonal mutations in each tumor sample (intra-tumoral clonal heterogeneity) and persistent mutations was assessed in the five cohorts treated with ICB (n = 524). **(a)** A moderate degree of correlation between the number of multi-copy mutations and decreased tumor clonal heterogeneity was only observed in melanoma. **(b)** Higher clonality tumors tended to have a lower number of only-copy mutations in the melanoma (Spearman ρ = -0.15, p = 0.03) and NSCLC-Shim cohorts (ρ = -0.15, p = 0.05), but no such difference was observed in the HNSCC (ρ = -0.23, p = 0.16), mesothelioma (ρ = -0.22, p = 0.18), and NSCLC-Anagnostou cohorts (ρ = 0.04, p = 0.74). **(c)** A significant correlation between pTMB and lower intra-tumoral clonal heterogeneity was observed in the melanoma cohort (Spearman ρ = 0.33, p = 1.7e-06). Spearman's rank correlation coefficients are shown, each tumor sample

represents a point and points are color coded based on tumor response on ICB. Two-sided p-values are calculated using asymptotic *t* approximation.

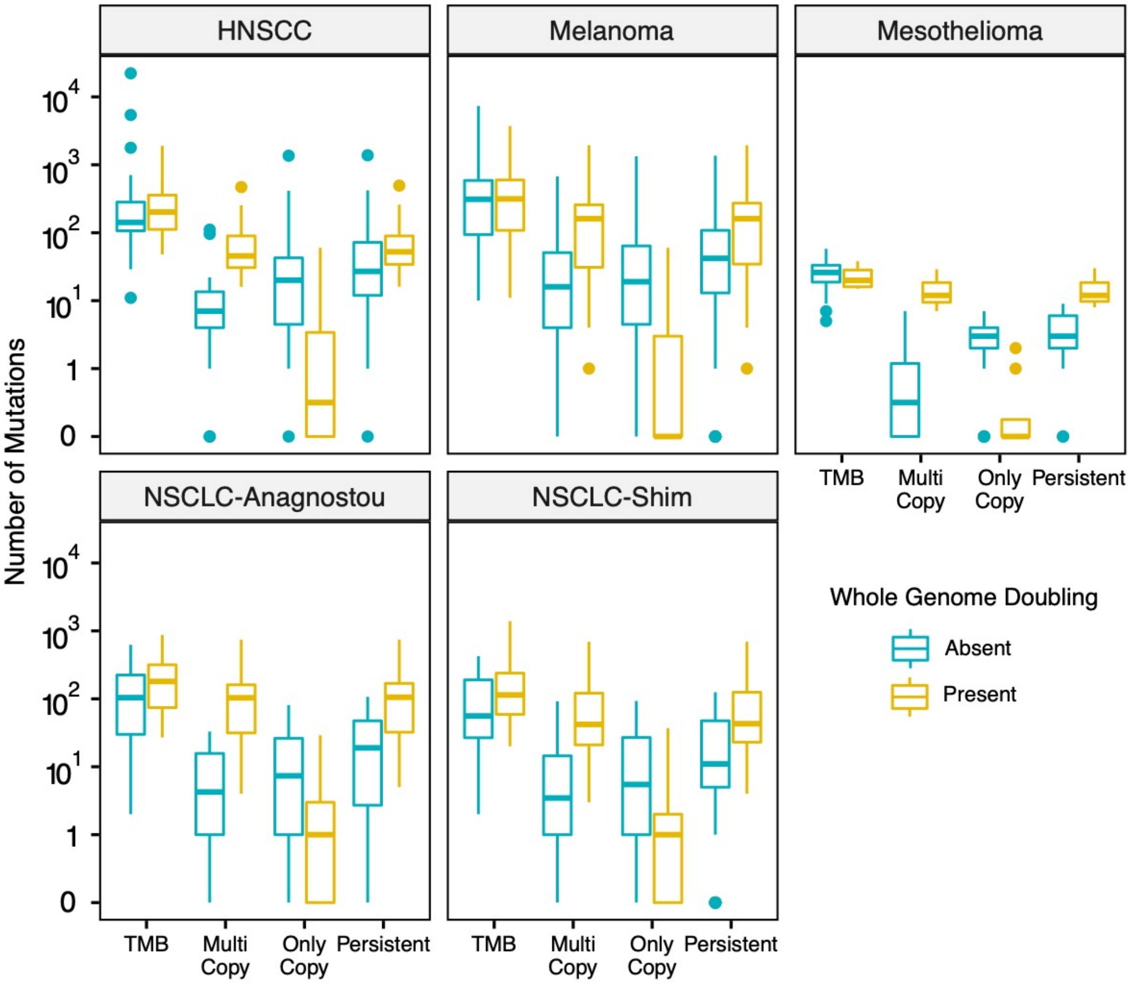

Supplementary Fig.2 Contribution of whole-genome doubling to acquisition of persistent mutations. TMB, the number of persistent mutations, mutations present in multiple copies per cell (multi-copy), mutations in single-copy regions (only-copy) of the genome were compared between tumors with and without evidence of whole genome doubling (WGD) across the five ICB cohorts (n = 524). In all cohorts analyzed, tumors with WGD harbored a significantly higher number of multi-copy mutations (HNSCC; p = 1.6e-05, melanoma; p = 3.14e-14, mesothelioma; p = 8.24e-06, NSCLC-Anagnostou; p = 6.82e-09, NSCLC-Shim; p = 9.3e-17) but a lower number of only-copy mutations (HNSCC; p = 5.23e-04, melanoma; p = 2.33e-19, mesothelioma; p = 6.13e-04, NSCLC-Anagnostou; p = 1.21e-03, NSCLC-Shim; p = 3.98e-09). HNSCC, melanoma, and mesothelioma tumors with and without WGD had similar levels of TMB (HNSCC; p = 0.58, melanoma; p = 0.76, mesothelioma; p = 0.45), while in the NSCLC-Shim cohort tumors with WGD also harbored a higher TMB (p = 5.91e-04). Tumors with WGD had significantly higher pTMB in melanoma, mesothelioma, and NSCLC, with a similar trend observed in HNSCC (HNSCC; p = 0.070, melanoma; p = 1.37e-06, mesothelioma; p = 1.84e-05, NSCLC-Anagnostou; p = 1.24e-05, NSCLC-Shim; p = 2.25e-08). Box plots depict the median value and hinges correspond to the first and third quartiles. The whiskers extend from the corresponding hinge to the furthest value within 1.5 * the interquartile range from the hinge.

Mann-Whitney U-test was used to compare values in tumors with and without WGD and calculate two-sided p-values.

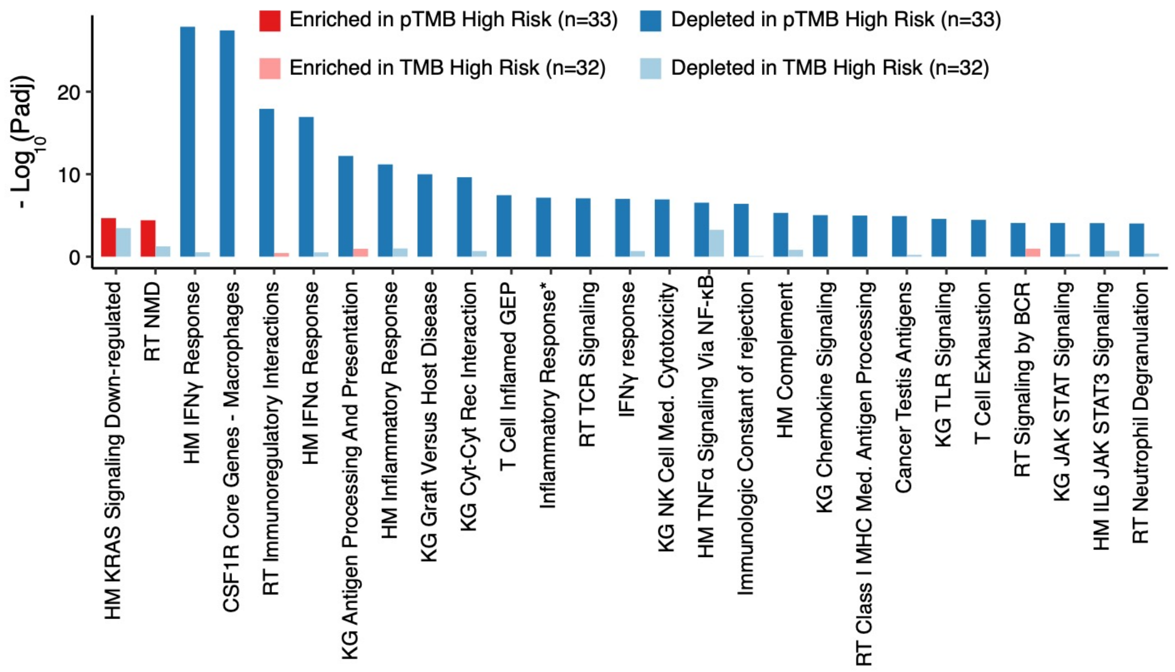

Supplementary Fig.3 Expression analyses of early stage melanomas in the TCGA cohort suggests a depletion of inflammatory pathways in high risk tumors as predicted by clonal pTMB. Gene set enrichment analyses of clonal pTMB-high risk (n=32) vs. clonal pTMB-low risk (n=63) melanomas highlight a significant under-representation of interferon-γ and inflammatory response gene sets in the microenvironment of tumors with pTMB-informed high vs low risk. In contrast, this pattern was not observed in tumors with TMB-informed high vs low risk. Pathways with a minimum adjusted p-value of 1e-05 in clonal pTMB high vs low risk comparison are shown. The T Cell Inflamed GEP gene set was derived from Cristescu et al., *Science*, 2018 and the Inflammatory Response gene set was derived by Ayers et al., *J Clin Invest*, 2017, while the remainder of gene sets were included in the Molecular Signatures Database (Methods). Nominal two-sided p-values are calculated using permutation testing and FDR adjusted p-values shown for gene set differential expression are provided for comparison of pTMB/TMB-high vs low risk groups. Abbreviations; HM: Hallmark, KG: KEGG, RT: Reactome, Cyt: Cytokine, Rec: Receptor, Med: Mediated.

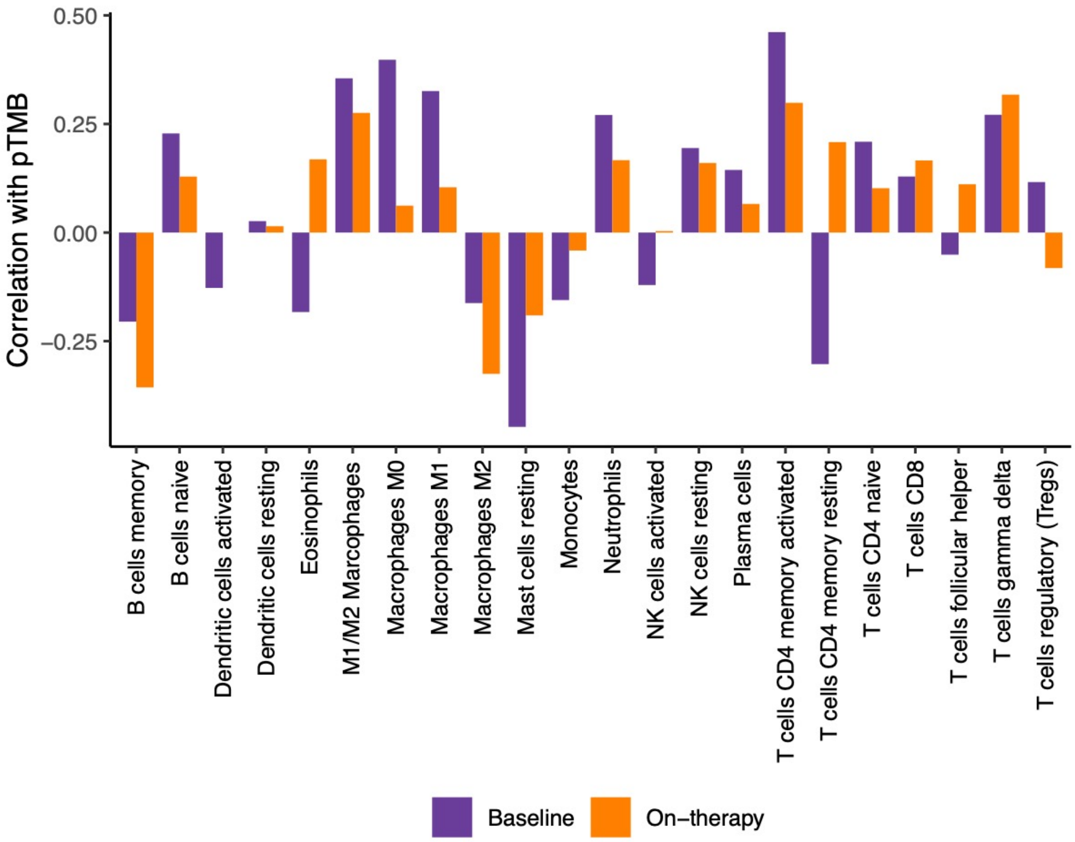

Supplementary Fig.4 Association of pTMB with abundance of immune cell subpopulations estimated by transcriptomic analysis of baseline and on-immunotherapy melanomas. CIBERSORT was used for deconvolution of RNA sequencing data and computation of the abundance of key immune cell subsets in the tumor microenvironment. Positive correlations were observed between pTMB CD4 and CD8 T cells as well as proinflammatory macrophages M1, while an anti-correlation was observed between pTMB and M2 macrophages. The ratio of M1 to M2 macrophages in baseline (n = 38, Spearman's ρ = 0.36) and on-therapy tumors (n = 31, Spearman's ρ = 0.28) was positively correlated with pTMB.

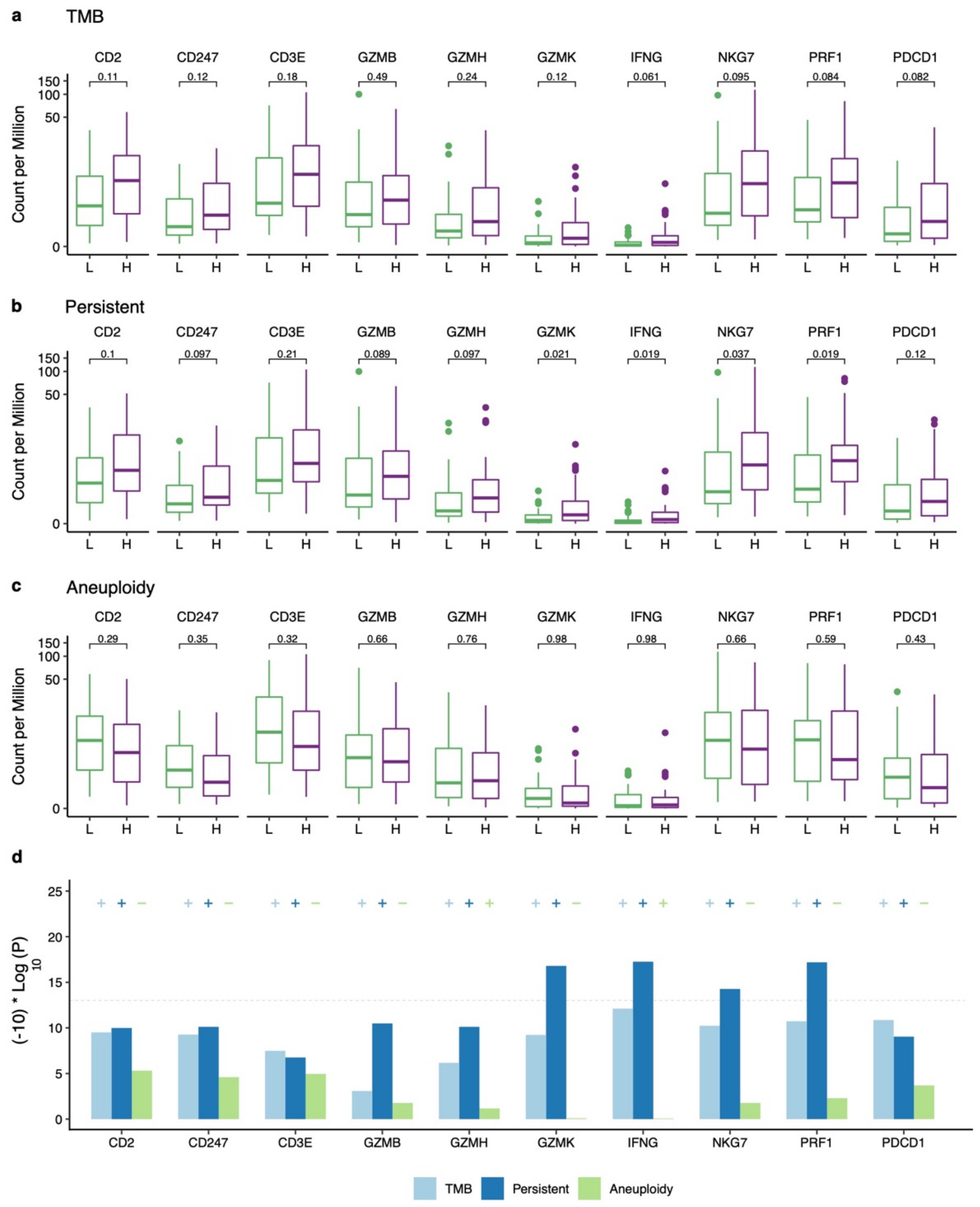

Supplementary Fig.5 Association of pTMB with cytolytic activity in melanoma. Expression of a selected set of genes indicative of cytolytic activity in TCGA melanoma tumors (n = 95). **(a)** No significant difference in expression was found in the TME of tumors in the top (H, n = 32) and bottom (L, n = 32) tertiles of TMB. **(b)** A higher level of cytolytic activity was observed in the TME of tumors with high (H, n = 32) vs low pTMB (L, n= 32), as indicated by the higher expression of GZMK , IFNG, NKG7, and PRF1 (MW U-test p = 0.021, 0.019, 0.04, and 0.019, respectively; two-sided test). **(c)** Tumor aneuploidy was not found to be a strong predictor of cytolytic activity in the set of analyzed tumors (CD2 p = 0.29, CD247 p = 0.35, CD3E p = 0.32, GZMB p = 0.66, GZMH p = 0.76, GZMK p = 0.98, IFNG p = 0.98, NKG7 p = 0.66, PRF1 p = 0.59, PDCD1 p = 0.43. MW U-test, two-sided). Box plots depict the median value and hinges correspond to the first and

third quartiles. The whiskers extend from the corresponding hinge to the furthest value within 1.5 * the interquartile range from the hinge. **(d)** pTMB predicted cytolytic activity in the TME more accurately compared to TMB and aneuploidy. Phred-scaled two-sided Mann Whitney U-test p-values are visualized. The annotated signs indicate the direction of association with cytolytic activity; i.e. a higher level expression in the top tertile group is marked as positive.

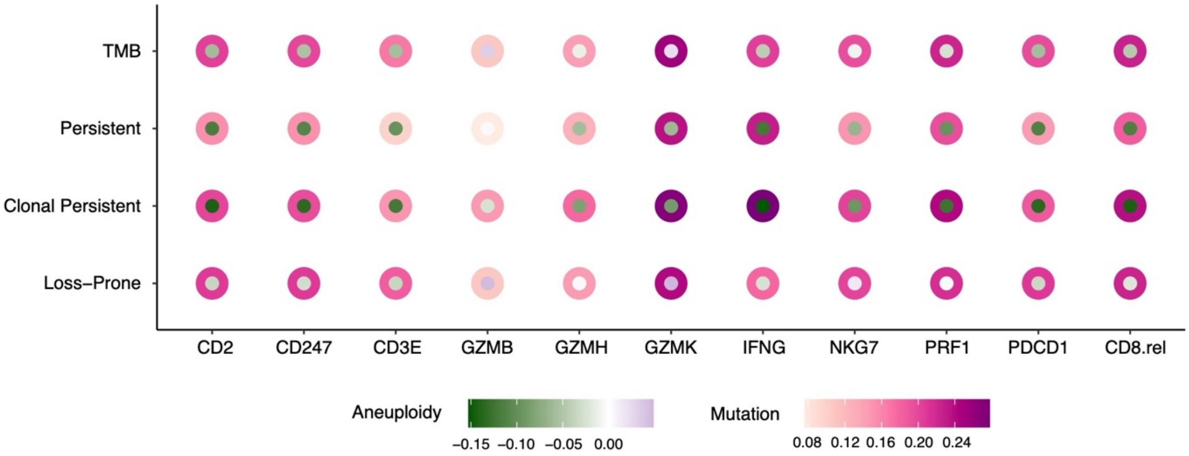

Supplementary Fig.6 Effects of pTMB and aneuploidy on cytolytic activity. Multivariate modeling of gene markers of cytolytic activity in the TME of TCGA melanoma tumors (n = 95) showed that high pTMB counteracted the negative (but non-significant) impact of aneuploidy and was positively correlated with cytolytic activity. A high value of pTMB and a low level of aneuploidy predicted higher expression of GZMK and IFNG (GZMK: $\beta_{pTMB} = 0.24$, $p = 0.03$; $\beta_{aneuploidy} = -0.06$, $p > 0.05$. IFNG: $\beta_{pTMB} = 0.23$, $p = 0.03$; $\beta_{aneuploidy} = -0.12$, $p > 0.05$; Two-sided p-values are calculated assuming normally distributed test statistic). CD8.rel: relative abundance of CD8 cells as estimated by CIBERSORT.
