# Supplementary material for: Persistent mutation burden drives sustained anti-tumor immune responses
Source: Nat Med. 2023 Jan 26;29(2):440–9. doi: 10.1038/s41591-022-02163-w (PMC9941047; doi:10.1038/s41591-022-02163-w)
Supplement: Supplementary file 2 — Reporting Summary [file 41591_2022_2163_MOESM2_ESM.pdf]

nature portfolio

Corresponding author(s): Valsamo Anagnostou
Last updated by author(s): Nov 23, 2022

Reporting Summary

Nature Portfolio wishes to improve the reproducibility of the work that we publish. This form provides structure for consistency and transparency in reporting. For further information on Nature Portfolio policies, see our Editorial Policies and the Editorial Policy Checklist.

Statistics

For all statistical analyses, confirm that the following items are present in the figure legend, table legend, main text, or Methods section.

n/a	Confirmed	
☐	☒	The exact sample size (*n*) for each experimental group/condition, given as a discrete number and unit of measurement
☐	☒	A statement on whether measurements were taken from distinct samples or whether the same sample was measured repeatedly
☐	☒	The statistical test(s) used AND whether they are one- or two-sided<br>*Only common tests should be described solely by name; describe more complex techniques in the Methods section.*
☐	☒	A description of all covariates tested
☐	☒	A description of any assumptions or corrections, such as tests of normality and adjustment for multiple comparisons
☐	☒	A full description of the statistical parameters including central tendency (e.g. means) or other basic estimates (e.g. regression coefficient) AND variation (e.g. standard deviation) or associated estimates of uncertainty (e.g. confidence intervals)
☐	☒	For null hypothesis testing, the test statistic (e.g. *F*, *t*, *r*) with confidence intervals, effect sizes, degrees of freedom and *P* value noted<br>*Give P values as exact values whenever suitable.*
☒	☐	For Bayesian analysis, information on the choice of priors and Markov chain Monte Carlo settings
☒	☐	For hierarchical and complex designs, identification of the appropriate level for tests and full reporting of outcomes
☐	☒	Estimates of effect sizes (e.g. Cohen's *d*, Pearson's *r*), indicating how they were calculated

Our web collection on statistics for biologists contains articles on many of the points above.

Software and code

Policy information about availability of computer code

Data collection	No software was used for data collection. The R package TCGAbiolinks (v2.25.0) was used to retrieve harmonized raw RNAseq counts data.
Data analysis	Statistical analyses were conducted using R (3.6). Bioinformatics analyses were performed using FASTP (v0.20.0), bowtie2 (v2.3.5), sambamba (v0.8), Strelka (v2.9.10), FACETS (v0.6.0), CIBERSORT (v1.06), and the R package fgsea (v1.20.0).

For manuscripts utilizing custom algorithms or software that are central to the research but not yet described in published literature, software must be made available to editors and reviewers. We strongly encourage code deposition in a community repository (e.g. GitHub). See the Nature Portfolio guidelines for submitting code & software for further information.

Data

Policy information about availability of data

All manuscripts must include a data availability statement. This statement should provide the following information, where applicable:

- Accession codes, unique identifiers, or web links for publicly available datasets
- A description of any restrictions on data availability
- For clinical datasets or third party data, please ensure that the statement adheres to our policy

Source data for the TCGA tumor samples were retrieved from http://cancergenome.nih.gov. WES-derived somatic mutation calls from the TCGA PanCancer Atlas MC3 project were retrieved from the NCI Genomic Data Commons (https://gdc.cancer.gov/about-data/publications/mc3-2017). Somatic copy number profiles (https://gdc.cancer.gov/about-data/publications/pancanatlas) and clinical data (https://gdc.cancer.gov/about-data/publications/PanCan-Clinical-2018) were accessed from Genomic Data commons. Previously published genomic data, re-analyzed here, were obtained from the material of the original publications and from dbGaP under accession code phs000452.v3.p17, and Sequence Read Archive (SRA) under accession codes SRP0958096, SRP0679388 and SRP0902948. WES sequence data for the HNSCC and NKI cohorts from patients who consented to data deposition can be retrieved from the European Genome-phenome Archive

(EGA accession number EGAS00001006660).
The de-identified clinical and genomic data used for the analyses in this study are available in the Supplementary Tables and all publicly available data elements have been referenced.

Field-specific reporting

Please select the one below that is the best fit for your research. If you are not sure, read the appropriate sections before making your selection.

☒ Life sciences ☐ Behavioural & social sciences ☐ Ecological, evolutionary & environmental sciences

For a reference copy of the document with all sections, see nature.com/documents/nr-reporting-summary-flat.pdf

Life sciences study design

All studies must disclose on these points even when the disclosure is negative.

Sample size	Tumor samples from the TCGA and 7 retrospective cohorts of patients treated with immune checkpoint blockade (ICB) were used in this study. The TCGA set comprised of 10,742 tumors, for which whole exome sequence data were analyzed with respect to their copy number profile and used to quantified the background rate of genomic loss. In 9,242 tumor samples, somatic mutation calls and copy number profiles were combined to characterize the prevalence of single-copy, multi-copy, and persistent mutations. The ICB cohorts comprised of a total of 524 patients, whose tumors were analyzed to determine the association between persistent tumor mutation burden and clinical outcome.
Data exclusions	In the TCGA cohort, tumor samples with missing copy number profile, somatic mutation calls, or clinical data were excluded.<br>In the ICB cohorts, tumor samples where the somatic copy number profile could not be resolved were excluded. In the Liu et al. melanoma cohort, samples with prior anti-CTLA4 treatment were excluded.
Replication	The study reports the proof-of-concept evaluation of a new genomic feature (persistent tumor mutation burden) in 10 independent cohorts treated with ICB. Sex was not considered in study design. Sex-stratified analyses were not performed as these were not within the scope of work presented. For the newly sequenced samples, self-reported sex is included in supplementary tables.
Randomization	The study was a retrospective cohort study, no randomization was performed.
Blinding	All data was de-identified.

Reporting for specific materials, systems and methods

We require information from authors about some types of materials, experimental systems and methods used in many studies. Here, indicate whether each material, system or method listed is relevant to your study. If you are not sure if a list item applies to your research, read the appropriate section before selecting a response.

Materials & experimental systems

n/a	Involved in the study	
☒	☐	Antibodies
☒	☐	Eukaryotic cell lines
☒	☐	Palaeontology and archaeology
☒	☐	Animals and other organisms
☐	☒	Human research participants
☒	☐	Clinical data
☒	☐	Dual use research of concern

Methods

n/a	Involved in the study	
☒	☐	ChIP-seq
☒	☐	Flow cytometry
☒	☐	MRI-based neuroimaging

Human research participants

Policy information about studies involving human research participants

Population characteristics	Population characteristics including self-reported sex and self-reported race are explicitly described in the manuscript. Sex or race-stratified analyses were not performed.
Recruitment	This is not applicable.
Ethics oversight	Sidney Kimmel Comprehensive Cancer Center Institutional Review Board, NKI Institutional Review Board, University of Chicago Comprehensive Cancer Center IRB 8980

Note that full information on the approval of the study protocol must also be provided in the manuscript.
